# Controlling Twisted Angles in Directly Grown MoS_2_ Bilayers via Tilt Grain Boundary Engineering

**DOI:** 10.1002/advs.202509280

**Published:** 2025-08-22

**Authors:** Xiaotian Li, Xuan Zhao, Luneng Zhao, Junfeng Gao, Zihan Zhao, Jian Yang, Tiantian Zhang, Yibiao Feng, Zejun Luo, Nan Liu, Jiacai Nie, Wenkai Zhang, Ruifen Dou

**Affiliations:** ^1^ Key Laboratory of Multiscale Spin Physics Ministry of Education School of Physics and Astronomy Beijing Normal University Beijing 100875 P. R. China; ^2^ Lab Material Modification Laser Ion & Electron Beams Dalian University of Technology Dalian 116024 P. R. China; ^3^ Beijing Key Laboratory of Energy Conversion and Storage Materials College of Chemistry Beijing Normal University Beijing 100875 P. R. China; ^4^ Applied Optics Beijing Area Major Laboratory Center for Advanced Quantum Studies School of Physics and Astronomy Beijing Normal University Beijing 100875 P. R. China

**Keywords:** chemical vapour deposition, moiré exciton, tilt grain boundaries, transition metal dichalcogenides (TMD), twisted bilayer

## Abstract

The moiré superlattices have garnered significant attention due to their unique twist‐angle‐dependent electronic and optical properties. Creating high‐quality twisted bilayer structures stands as one of the major frontiers in the study of correlated moiré physical properties, however, which remains a challenge. Here, a cyclical‐carrier‐gas chemical vapor deposition method is employed to grow high‐quality twisted bilayers MoS_2_. Stacking configurations and the growth mechanism of twisted bilayer MoS_2_ are systematically investigated, revealing that the relative rotation angle between the two layers is guided by the tilt grain boundaries (GBs) of the bottom layer. This relationship is elucidated through real‐time monitoring of the formation process of the top layer from nucleation to coalescence. Meanwhile, the interlayer exciton (X^I^) strongly couples to the twist angle, and the enhanced intensity of X^I^ peaks in TB‐MoS_2_ at specific twist angles of ≈22° and 38° is evidenced by low‐temperature (10K) photoluminescence and second harmonic generation spectra, which can be ascribed to the X^I^ localization in the periodical moiré superlattice. The findings provide a viable method for the in‐ situ preparation of layered twisted materials guided by tilt GBs, which facilitates the exploration of novel optical and excitonic physics in moiré systems.

## Graphical abstract

In this study, the stacking configurations and the growth mechanism of twisted bilayer (TB) MoS_2_ are systematically investigated, revealing that the relative rotation angle between the two layers is guided by the tilt grain boundary (GBs) of the bottom layer. This relationship is elucidated through real‐time monitoring of the formation process of the top layer from nucleation to coalescence. Meanwhile, the interlayer exciton (X^I^) strongly couples to the twist angle and enhanced intensity of X^I^ peaks in TB‐MoS_2_ at specific twist angles of ≈22° and 38° are evidenced by low‐temperature (10K) photoluminescence and second harmonic generation spectra, which can be ascribed to the X^I^ localization in the periodical moiré superlattice. The findings first elucidate the growth mechanism of tilt angle guiding the top layer growth for in situ preparation of layered twisted materials, which facilitates the exploration of novel optical and excitonic physics in TMD‐based moiré systems.

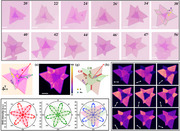

## 1 Introduction

Man‐made superlattices through overlaying 2D layered materials with misaligned interlayer twists or lattice mismatches generate large periodical moiré patterns, undeniably presenting novel opportunities for investigating the electron–electron interactions in 2D layered materials.^[^ ^1^, ^2^, ^3^, ^4^, ^5^, ^6^, ^7^, ^8^, ^9^, ^10^ ^]^ Twisted bilayer (TB) graphene, especially magic‐angle graphene, has emerged as an alluring material for the investigation of twistronics because of its unique twist‐angle‐dependent properties such as correlated Mott‐insulator and superconductivity.^[^ ^11^, ^12^, ^13^, ^14^ ^]^ Beyond TB graphene, the bilayer transition metal dichalcogenides (TMD) with a relative twist angle between them have generated great interest due to their emerging mini‐flat band structures and novel excitonic properties modulated by the periodical moiré pattern.^[^ ^15^, ^16^, ^17^, ^18^, ^19^, ^20^, ^21^ ^]^ Due to its higher orbital degeneracy and resultant existence of the topologically nontrivial quantum state in TB TMD homobilayer, the quantum anomalous Hall effect was realized in a twisted MoTe_2_ bilayer homostructure by applying an extremely large external electric field.^[^ ^22^, ^23^ ^]^ Meanwhile, the excited states of interlayer and intralayer excitons can be effectively manipulated by the moiré superlattice in twisted TMD‐based homobilayers with locally symmetric atomic reconfigurations.^[^ ^24^, ^25^, ^26^ ^]^ To date, many efforts have been devoted to the preparation of high‐quality TB TMD‐based homostructures from the perspective of fundamental research and practical application. Experimentally, the above fabrication approaches can be roughly divided into two strategies: the top‐down exfoliation and the bottom‐up synthesis.^[^ ^27^, ^28^ ^]^ The top‐down approach, such as the tear‐and‐rotate involved in the adhesive tape‐assisted dry transfer and the liquid‐assisted wet transfer, allows for the preparation of van der Waals moiré superlattices with interlayer twist angles. However, uncontrollable shifts in the angle lead to poor uniformity in the moiré angle distribution as well as the susceptibility to contamination of the interface, which frequently occurs when individual homobilayers of TMDs are stacked together via the tear‐and‐rotate approach. Consequently, the experimental uncertainties associated with the top‐down approach have considerably hindered in‐depth studies of twist angle‐dependent excitonic properties in twisted TMD‐based homobilayers.^[^ ^21^, ^26^ ^]^ To overcome the limitation of the top‐down exfoliation, the direct growth of twisted TMD‐based homobilayer via chemical vapor deposition (CVD) is currently considered as one of the most promising methods to acquire the twisted structures with the uniform angle distribution and the excellent interface.^[^ ^29^, ^30^, ^31^ ^]^ However, deliberately and accurately controlling the twist angle in layered structures is still tough work, since twisted configurations are almost thermodynamically unfavorable during the direct growth. Meanwhile, the fundamental issues existing in the direct growth of twisted structures, mainly including reproducible growth conditions and understanding the growth mechanism, have not yet been intensively addressed. Accordingly, developing a reproducible CVD route to obtain the high‐quality twisted bilayer TMD structures is unambiguously required to understand the beneath growth mechanism and further intensively explore the twist angle‐dependent interlayer interactions and excitonic properties.

In this contribution, high‐quality TB MoS_2_ structures with a wide range of twist angles from 20° to 60° were fabricated on SiO_2_/Si substrates via a feasible CVD method associated with a cyclically changing the carrier Ar gas. Polarization‐resolved second harmonic generation (SHG) enables precise identification of the twist angles between the neighboring layers in TB MoS_2_. The growth process of the TB MoS_2_ is visualized through real‐time monitoring the nucleation and coalescence of the top layer, demonstrating that the top layer rotated a twist angle related to the bottom layer is triggered by the tilt grain boundary (GB) of the bottom layer. Variable temperature photoluminescence (PL) spectroscopy studies on the TB MoS_2_ structures indicate that the indirect exciton (X^I^) peak shifts in‐red with changing the twist angle, which is consistent with the variation of the indirect band gap with the twist angles calculated by density functional theory (DFT). Moreover, low temperature (10K) PL results show that the X^I^ strongly couples with special twist angles, in which the intensity of the X^I^ is enhanced in the as‐grown TB MoS_2_ at commensurate angles of ≈22° and 38°. Correspondingly, the second harmonic generation (SHG) spectra from the TB‐MoS_2_ at around 22° and 38° exhibit an increased intensity at the energy of 1.5 eV. This means that moiré superlattice provides the periodical quasi‐quantum potentials to tune the X^I^ with the enhanced nonlinear optical properties. Our work opens up a feasible way to directly grow highly crystalline TMD‐based TB homostructures for further tuning their energy band structures and the correlated excitonic properties.

## 2 Results and Discussion

### 2.1 Growth of TB‐MoS_2_ Homostructure with a Wide Range of Twist Angles

**Figure 1:**
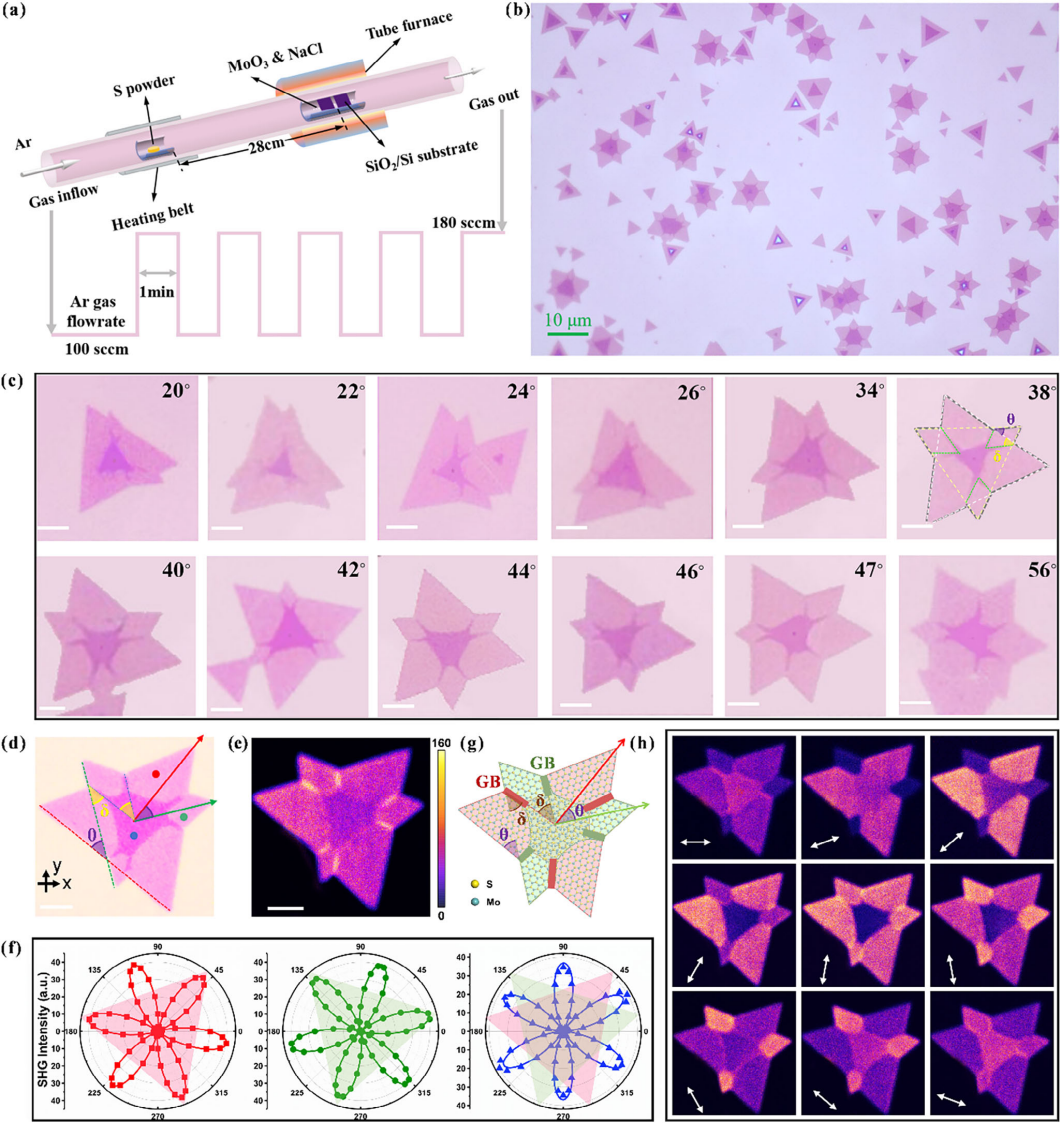
The CVD growth of TB‐MoS_2_ crystals with various twisted angles from 20° to 56°. a) A CVD setup and the carrier gas (Ar) changing rate during the whole growth process. b) A large‐scale OM image of the as‐grown TB‐MoS_2_ crystals with different twisted angles on a SiO_2_/Si substrate. c) The representative OM images of TB‐MoS_2_ homostructures with interlayer angles from 20° to 56° (± 0.5°). The scale bar is 3 µm. d, e) A representative OM image and the SHG intensity mapping image of the sample of TB‐MoS_2_ at 38°, Red and green points indicate monolayers with different orientations, while the blue point indicates the twisted bilayer region. f) Polar plots of parallel components of SHG intensity of 38°‐TB MoS_2_. The polar plots from left to right are measured in the same color region as marked by the red, green, and blue dots in (d), respectively. g) A schematic structure of TB MoS_2_ with a twist angle of θ between the two layers and a tilt GB angle of δ between the two tilt GBs. h) A series of polarization‐resolved SHG mapping images was detected from 38°‐TB MoS_2_ under excitation at λ_ex_ = 808 nm. White arrows indicate the polarization orientation of the analyzer.

To obtain bilayer MoS_2_ with different twist angles, we design key growth parameters mainly including cyclically changing the carrier Ar gas to overcome the high formation energy of stacking the top layer twisted from the bottom layer on SiO_2_/Si substrates during the CVD process. The CVD setup and the carrier gas (Ar) change rule are shown in **Figure** **1**a. The more detailed growth parameters including the ratio of two precursors of Mo and S, the substrate temperature and the growth interval are provided in Method. Figure 1b shows a typical optical microscopy (OM) image, illustrating that large‐scale TB MoS_2_ homostructures with a wide range of twist angles can be successfully synthesized by using a feasible CVD approach. The representative OM images from TB MoS_2_ with twist angles from 20° to 56° are institutively displayed in Figure 1c and Figure S1 (Supporting Information), as well as scanning electron microscopy (SEM) images supplied in Figure S2 (Supporting Information). It is well‐known that there is a distinct relationship between the shape of monolayer MoS_2_ grown by CVD and its crystal orientation,^[^ ^31^, ^32^ ^]^ thus the interlayer twist angle (θ) between the top and the bottom layer is defined according to the relative direction of the triangle edges of the two layers, depicted in the OM image of a 38°‐TB MoS_2_ pattern (Figure 1c). The interesting phenomenon is that the bottom layer for the as‐grown TB MoS_2_ structures shows a quasi‐six‐point star shape (sometimes one or two arms missing) with a tilt angle (δ) between two misoriented triangular domains (two green dashed lines), as highlighted in the OM image of 38°‐TB MoS_2_ of Figure 1d. This quasi‐six‐point star MoS_2_ structure is analogous to the regular six‐point star shape composed of six twin mirror grains connecting by a tilt angle of 60°, as shown in Figure S3a (Supporting Information). In this quasi‐six‐point star MoS_2_, the tilt angle is formed owing to the connection of two rotational neighboring domains.^[^ ^33^, ^34^, ^35^ ^]^ Accordingly, the tilt angle between the two neighboring boundaries in the quasi‐six‐point star structure can be calibrated to be ≈38°, perfectly coincident with the twisted angle (θ) between two neighboring layers. As exampled by the 38°‐TB MoS_2_, all as‐grown TB MoS_2_ patterns with a wide range of twist angles exhibit the same characteristic, that is, the twist angle between two neighboring layers equaling the tilt angle of two tilt grain boundaries (GB), as shown in Figure 1d and Figure S1 (Supporting Information). This means that the growth of the top layer is guided by the tilt GB, presenting the twist angle overlapping with the tilt angle, which can be evidenced by the SHG.

SHG, being highly sensitive to structural symmetry, is deemed as an effective method for the precise determination of twist angle between the two layers.^[^ ^36^, ^37^ ^]^ Here, we systematically explore the relationship between the twist angle and the tilt angle from the two GBs. The SHG signal has been confirmed through fundamental characterizations, including wavelength verification and power dependence analysis of the SHG signal intensity, which are provided in the Method and Figure S4 (Supporting Information). To evaluate the twist angle between the two layers, we conduct SHG measurements for all as‐grown TB MoS_2_ samples. Figure 1e shows a representative SHG mapping image of a 38°‐TB sample acquired under linear polarization excitation without an analyzer before the detector. This 38°‐SHG image, corresponding to the OM image in Figure 1d, distinctly demonstrates a misaligned bottom layer and a twisted top layer. Moreover, the two misaligned triangles in the bottom layer are divided into six asymmetric petals by six bright tilt GBs. To calibrate the tilt orientation between two misaligned triangles, the polarization‐resolved SHG is performed to detect different grains as marked by the red and green colored dots in Figure 1d. The corresponding SHG intensity signals are presented in the left and middle polar plots of Figure 1f, indicating distinct six‐fold symmetric patterns with the azimuthal direction of SHG oriented along the crystalline symmetric axis. Obviously, both polar plots display a 38.4° angular offset between their individual azimuthal angles, which directly corresponds to the relative misaligned orientation angle of 38.4° between two neighboring crystalline symmetric axes. This means that the presence of a tilt angle between the two twin tilt GBs (38.4°), as highlighted a δ by the yellow colored dashed lines in Figure 1d. The blue SHG polar plot obtained in the blue colored dot corresponding to the bilayer MoS_2_ region is shown in the right panel of Figure 1f, further indicating a six‐fold symmetry SHG plot. The twist angle between the top and the bottom layers can be determined by the SHG signals measured from the top and the bottom layer, respectively.^[^ ^36^, ^37^ ^]^Through comparing the azimuthal orientation of the blue and the green polar plot, we find that the angular offset is ≈19.3°. Previous researches show that the angular offset determined by the SHG polar plot is half of the twist angles between the top and the bottom layer.^[^ ^38^, ^39^ ^]^ Accordingly, the interlayer twist angle between the top and the bottom layer can be calculated to be 38.6°, double the angular offset of 19.3°. Here, this geometric relationship between the interlayer twist angle and the angular offset determined by the SHG polar plots in the TB MoS_2_ structure is shown in the schematic structure in Figure 1g in detail, being well reproducible with that determined by the triangular edges imaged from OM in Figure 1c. More SHG polar plots measured in other as‐grown TB MoS_2_ samples are shown in Figure S5 (Supporting Information), with the calculated twist angles listed in Table S1 (Supporting Information). The above SHG results directly provide evidence that the interlayer twist is coincident with the tilt angle between two twin GBs in the bottom layer.

To clearly distinguish the tilt GBs of the misaligned bottom layer and demonstrate the uniformity for all the as‐grown TB MoS_2_, the polarization‐resolved SHG intensity mapping analysis across the whole sample area is obtained by rotating polarization analyzer, as shown in Figure 1h and Figure S6 (Supporting Information). The arrows indicate the analyzer orientation. Through continuously rotating the analyzer within the angle range from 0° to 180°, swapping at a 20° per step, a series of representative SHG mapping images from 38°‐TB MoS_2_ are obtained in Figure 1h, revealing the intensity‐modulated SHG signal in different petals on the same TB MoS_2_ sample. The polarized SHG signals strongly depend on their respective orientation of the grains, illustrating that the orientation of the tilt grains is determined by the tilt angle (δ). Moreover, the SHG intensity mapping images prove the growth tendency of the top layer along the GBs of the bottom layer through the observation of the crystal whiskers from the top layer along the GBs.

### 2.2 Growth Mechanism of TB‐MoS_2_ Guided by Tilted GBs

**Figure 2:**
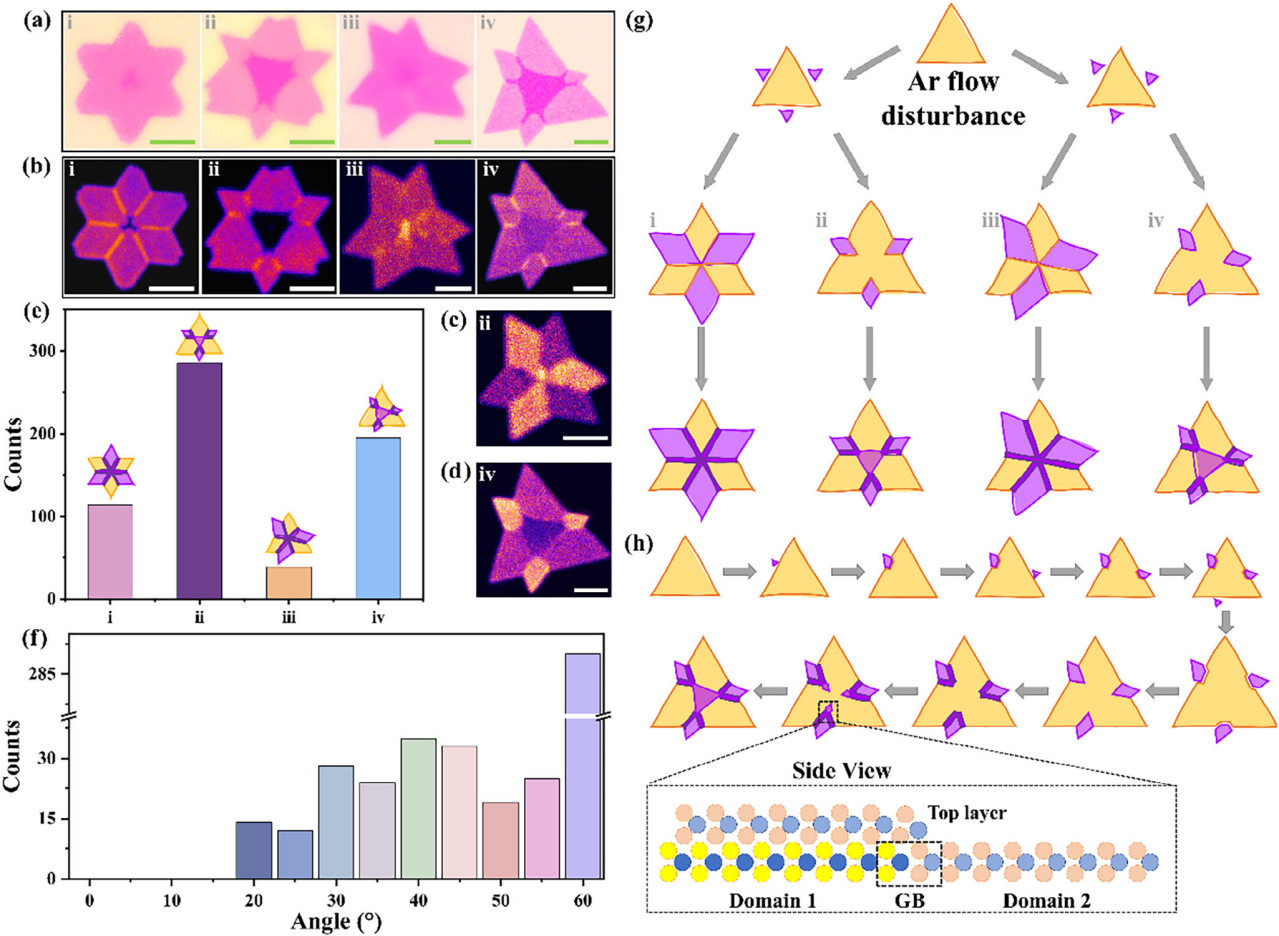
The growth process and a schematic model of TB MoS_2_ induced by multi‐twin grain‐structured bottom layer. a,b) Representative OM images and SHG mappings of the four structural types: high‐symmetry monolayer (i), unequal bilayer (ii), tilted monolayer (iii), and tilted bilayer (iv). c,d) Polarization‐resolved SHG mapping of tilted configurations (types iii and iv), revealing alternating intensity contrast that directly resolve non‐60° crystallographic misalignment. e) Statistical distribution of the four six‐point‐star MoS_2_ sample types. f) Twist angle distribution histogram of TB MoS_2_. g) Growth model under cyclic gas‐flow CVD resulting in four distinct forms of six‐point‐star MoS_2_ samples h) Schematic model of the bottom tilt six‐point‐star structure and the top layer growth along the GBs from the bottom‐layer domains. The illustration is the side view of the top layer along the GBs.

To elucidate the formation mechanism of TB MoS_2_ during the cyclical gas‐flow CVD, with a focus on the role of tilted GBs in the bottom layer and their influence on the nucleation and growth of the top layer, we performed a systematic analysis of the morphological evolution of six‐point‐star motifs under controlled gas flow perturbations. Through extensive morphological characterization, we identified four distinct types of six‐point‐star structures: i) a high‐symmetry six‐point‐star monolayer with a top‐layer nucleation center,^[^ ^40^, ^41^, ^42^, ^43^ ^]^ ii) an asymmetric bilayer star, iii) a tilted monolayer star with a top‐layer nucleation center, and iv) a tilted bilayer star. Representative OM images and SHG mapping images of the four six‐point‐star samples are shown in **Figure** **2**a,b, respectively. The high‐symmetry six‐point‐star monolayer (type i) is very normal during the CVD growth with a constant gas flow. With a top‐layer nucleation center, the top layer normally exhibits a similar azimuthal orientation related to the bottom layer. As a result, an AA and AB stacking MoS_2_ bilayer is alternatively formed.^[^ ^40^ ^]^ For the bilayer configurations (types ii and iv), the bottom layer is formed by the coalescence of a central triangular domain with three quais‐rhombic satellite domains. During the coalescence process, the interfacial interactions between these domains generate GBs that serve as guiding templates for subsequently assembly of the top layer, ultimately forming the bilayer structure. For tilted configurations (types iii and iv) of the bottom layer, twin grains with non‐0° and 60° tilted angle easily form, indicating the gas‐flow perturbations during the CVD growth. As a result, the top layer with a twist angle relative to the bottom layer can be generated. These findings highlight the critical role of gas flow dynamics and GB interactions in determing the morphological evolution of the monolayer and bilayer MoS_2_. Crystallographic misalignment in monolayer and bilayer MoS_2_ is directly evidenced by polarization‐resolved SHG mapping images in Figure 2c,d, respectively. Here, alternating SHG intensity contrasts between neighboring tilted domains reflect angular deviations from ideal alignment, further suggesting that the top layer rotates an angle relative to the bottom layer guided by the tilt angle of two twin GBs. In addition, these non‐60° tilt angles induce asymmetric domain collisions, possibly leading to the formation of strained GBs. Polarized SHG plots from the tilt GBs reveal the existence of compressive deformation, as shown in Figure S7 and Table S2 (Supporting Information), with the compression direction oriented approximately perpendicular to GBs. This strain originates from structural rearrangement near the tilted GBs of the bottom layer, as confirmed by atomic‐resolution scanning transmission electron microscopy (STEM). High‐angle annular dark field STEM (HAADF‐STEM) imaging directly reveals compressive lattice distortion at the GB region, as shown in Figure S8 (Supporting Information), in which twin mirror atomic registration is directly resolved. These results prove the GB formation in the bottom layer. Consequently, the top layer preferentially nucleates and propagates along these unstable tilted GBs,^[^ ^33^, ^34^, ^35^ ^]^ resulting in characteristic twist angles relative to the bottom layer. The corresponding structural evolution model in Figure S9a (Supporting Information) provides direct visualization of this tilted‐GB‐guided top‐layer formation mechanism. In experimental, Figure S9b,c (Supporting Information) explicitly demonstrates the formation process of the top MoS_2_ layers with 60° and 58° twist angles, respectively, progressing from nucleation at the bottom‐layer center through domain expansion along tilted GBs to complete top monolayer coalescence (Stages S1‐S5).

By integrating OM and SHG analyses, we demonstrate that airflow perturbations introduce two synergistic modifications to bottom‐layer domains: i) size disparities among neighboring domains and ii) angular deviations from ideal crystallographic alignment (60°). The interplay of these two factors creates preferential nucleation sites along misoriented GBs, enabling deterministic formation of TB MoS_2_ configurations. To statistically validate this mechanism, we analyze 633 characterized samples. The resulting population distribution reveals a distinct hierarchy, as shown in Figure 2e: asymmetric bilayer stars (type ii constitute the majority (45.0%) followed by tilted bilayer stars (type iv, 30.8%) and high‐symmetry monolayer six‐point stars (type i, 18.0%), and tilted monolayer stars (type iii, 6.2%). This distribution suggests that gas flow disturbance primarily modulates the domain size and induces the production of the bilayer structure with an interlayer twist.

Figure 2f shows the twist angles of a six‐point star bilayer spanning from 20° to 60°, with a dominant peak at 60° and a secondary peak near 40°, while angles below 20° are rarely observed. This distribution can be attributed to the strain relaxation at large twist angles, facilitating by the incorporation of atoms at the GBs. TB MoS_2_ with a twist angle smaller than 20° is less stable, which is consistent with the previous studies.^[^ ^31^, ^44^ ^]^ Figure 2g proposes a hierarchical model of the growth of six‐point‐star monolayer and bilayer during the cyclic gas‐flow CVD process.

The growth process begins with a small triangular grain, followed by the deposition of new grains at the midpoints of the initial triangle edge. Under stable gas flow conditions^[^ ^33^ ^]^, capillary forces enforce the new grain to orient a 60° alignment relative to the initial triangle for minimizing the interfacial energy. However, under turbulent carrier gas flow, the micro‐growth surrounding is changed continuously, which feasibly introduces the orientation deviation between the neighouring grains to form the tilted grains. As the grains extend their competitive growth triggers two distinct engulfment modes, as shown in Figure 2g,i) complete engulfment, where GBs penetrate into the symmetric center of the initial triangular, separating them into six symmetric domains, and ii) partial engulfment, in which the initial grain remains with the three edges decorated by three tiny wing‐like grains. During subsequent growth, three wing‐like grains propagate along GBs, to ultimately generate the top layer. This framework offers a reasonable explanation for the formation of all four six‐point‐star configurations (i–iv in Figure 2a,g). Specially, the dynamic formation process of TB MoS_2_ (type iv) is displayed in Figure 2h and Movie S2 (Supporting Information), directly demonstrating the domain evolution in the six‐point star monolayer and the top layer rotation growth relative to the bottom monolayer. The side‐view schematic structure in the bottom panel of Figure 2h unveils a critical geometric relationship between the top layer and the bottom layer. It shows that the top layer is guided by the GBs in the bottom layer.

### 2.3 Resonant Enhancement of X^I^ in TB‐MoS_2_ at commensurate angles

**Figure 3:**
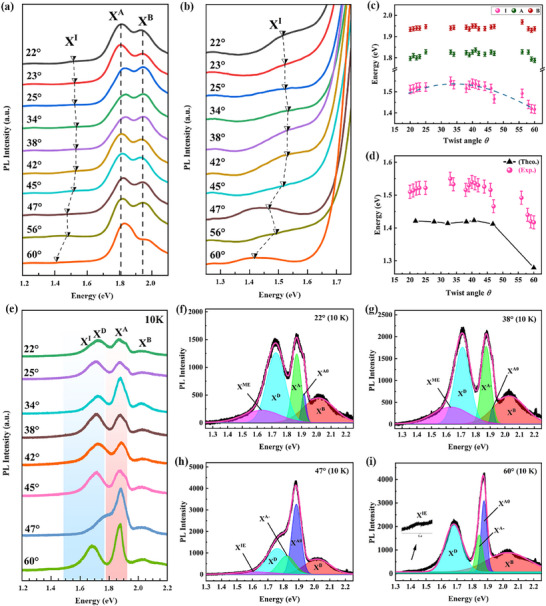
PL spectra of TB MoS_2_ with a wide range of twist angles measured at RT and LT (10K). a) RT PL spectroscopy data in samples of TB MoS_2_ homostructures with different twisted angles. b) Local zoom‐in PL spectra of TB MoS_2_ homostructures showing the interlayer excitons changing with the twist angles. c,d) The relationship of the energy positions of A exciton (X^A^), B exciton (X^B^) and indirect interlayer exciton peak (X^I^) with the twist angle. Among (d), the indirect band gaps of the TB MoS_2_ homosturctures with various twist angles calculated by DFT (the black triangular line) fit well the optical band gap measured by PL spectroscopy. e) PL spectroscopy data at low temperature (10K) obtained in samples of TB MoS_2_. The used laser power is 0.2 mW. f–i) LT PL spectrum and the corresponding Gaussian fitted peaks of TB MoS_2_ with the twist angle of 22° (f), 38° (g), 47° (h), and 60° (i), respectively. The inset of (i) with the black arrow is a zoomed‐in view of the spectrum near 1.42 eV, showing the interlayer exciton peak.

To verify the crystal quality of the as‐grown TB MoS_2_, Raman spectroscopy is further performed for all as‐grown MoS_2_ with a wide range of angles in Figure S10 (Supporting Information). The optical properties of the TB‐MoS_2_ homostructures with various twist angles were investigated via PL spectroscopy at room temperature (RT) and low temperature (LT) (10K). As displayed in **Figure** **3**a,b, a series of RT PL spectra from the TB‐MoS_2_ homostructures contain three peaks of A exciton (X^A^), B exciton (X^B^), and indirect interlayer exciton peak (X^I^). For the 60°‐TB MoS_2_ pattern, these peaks of X^A^, X^B^, and X^I^ are located at ≈1.86, 1.94, and 1.42 eV, respectively. The X^A^ exciton arises from direct excitonic transitions between the conduction band minimum (CBM) and the valence band maximum (VBM), which is a direct K‐valley exciton in the first Brillouin zone of MoS_2_ crystal.^[^ ^45^, ^46^ ^]^ The X^B^ exciton is derived from direct excitonic transitions between the CBM and the split valence band due to the spin‐orbit coupling.

The X^I^ is induced by the transition between the valence band maximum (Γ point) and the conduction band minimum (K or Q point) in the K space. The PL emission energies of the X^A^ and X^B^ exciton is slightly sensitive to the twist angle, where the X^A^ and X^B^ as intralayer excitons from the TB MoS_2_ samples at twist of ≈20°–50° exhibit a slight red shift compared with that of TB MoS_2_ at the twist of 0° and 60°, as shown in Figure 3c, which is in a good agreement with previous studies.^[^ ^45^, ^46^ ^]^ Obviously, the energy positions of X^I^ peaks exhibit a non‐monotonic evolution with twist angles, originating from the interlayer coupling tuned by the twist angle, which leads to the energy of critical points Q (Γ) downshift or upshift as the twist angle is changed. The present experimental result is in well consistent with our DFT calculations, as shown in Figure 3d. We systemically calculate the energy band structures of the bilayer MoS_2_ with twist angles of 21.8°, 27.8°, 32.2°, 38.2°,40.7°, 46.8°, and 60°, where atomic registration structures and energy band structures of bilayer MoS_2_ with the twist angle were drawn in Figures S11, S12 (Supporting Information). The geometrical structures display the yellow solid lines remarkably highlighting the moiré supercell. The moiré wavelength is given as *L* ^M^ =a/2sin(θ/2), where *a* is the in‐plane lattice constant of the monolayer MoS_2_ and the size of the moiré supercell is strongly related to the twist angle. The calculated indirect Γ‐K band gaps of TB MoS_2_ homostructures with different twist angles suggest the salient dependence of the indirect band gap on the corresponding twist angles. The most stable 2H (60°) stacking bilayer MoS_2_ shows a Γ‐K band gap (1.279 eV) much lower than that of other bilayer MoS_2_, which is in agreement with our experimental result. Meanwhile, the TB‐MoS_2_ homostructures with commensurate twist angles of 21.8°, 27.8°, 32.2°, 38.2° hold the indirect bandgap ranging from 1.412 to 1.421 eV slightly lower than that (1.423 eV) of other twisted MoS_2_ bilayer at the incommensurate angle of 40.7°, indicating the relatively strong interlayer coupling from the commensurate moiré superlattice compared to incommensurate moiré superlattice.

To further understand the excitonic properties of as‐grown TB MoS_2_ structure with various moiré patterns, the low‐temperature (LT) PL spectra at T = 10 K are comparably performed for samples of TB MoS_2_ homostructure at twist angles of 22°, 25°, 34°, 38°, 42°, 45°, 47°, and 60°, as shown in Figure 3e. Obviously, the peaks of the X^A^ and the X^B^ exciton at the energy of ≈1.84 and 1.95 eV in LT PL spectra of TB‐MoS_2_ homostructures at commensurate angles of ≈22°, 25°, 38° and 45° present a wide full width at half‐maximum, which might induced by the excitonic complexes. Moreover, Figure 3e–h show two additional new peaks located at ≈1.62 and 1.75 eV on the left side of the A exciton peak, respectively identified in the LT PL spectra from TB MoS_2_ sample at 22°, 25°, 38° and 45°. Among them, one excitation peak projected at ≈1.75 eV might be related to the defect emission (X^D^)^[^ ^47^ ^]^, originating from the localized excitons bound by defects such as grain boundary generated due to the tilt grain boundaries guiding the growth of bilayer MoS_2_ during the CVD growth procedure.

As for another peak at the energy in the range of 1.58–1.62 eV on the left side of the X^D^ emission peak in the LT PL spectra, the radiation recombination can be assigned to the X^I^ similar to that in PL spectra measured at room temperature. At low temperature, the energy peak of X^I^ shifted to the high energy position from ≈1.44 to 1.58 eV. Moreover, the intensity of X^I^ was obviously manipulated by the twist angle. Considering the moiré superlattice resulted from the commensurate twist angle holding the great moiré potential, the excitons including the interlayer and the intralayer excitons may be tuned and confined in the special atomic sites with the deep moiré potential to give rise to moiré excitons (X^IE^).^[^ ^21^, ^48^ ^]^To better ensure the twist‐angle dependent moiré excitons, the intensity ratio of the X^IE^ and the X^A^ of the TB MoS_2_ is calculated by the function of $R_{IE/A}=I_{X^{IE}}\;/I_{X^{A}}$. It is clearly found that the *R*_*IE*/*A*_value corresponding to the commensurate superlattice of 38°‐TB MoS_2_ is the largest, near to 1.06 (Table S3, Supporting Information), among the as ‐grown TB MoS_2_ at twisted angles of 25°, 32°, 33°, 34°, 38°, 41°, 42° and 45° (see PL spectra in Figure S13, Supporting Information). This means that interlayer excitons can be bound by the deep moiré potential in the different high symmetric sites in the moiré superlattice, which only happens on the commensurate superlattices caused by the commensurate large twist angles. The previous calculated deep moiré potential can be analogous to the binding energy of interlayer excitons of ≈100 eV, predominantly resulting from the miniband in refolded Brillouin zones.^[^ ^21^, ^33^ ^]^. Meanwhile, the *R*_*IE*/*A*_ value of 22°‐TB MoS_2_ is 0.42 larger than that of TB‐MoS_2_ structures at incommensurate angles (Table S3, Supporting Information). The above analysis claims that X^I^ can be trapped in high‐symmetricatomic‐registration sites, as shown in schematic structures in Figure S12 (Supporting Information), resulting in the energy shift and increased intensity in LT PL spectra.

### 2.4 Twist‐Angle‐Dependent Exciton Coupling Proved by SHG

**Figure 4:**
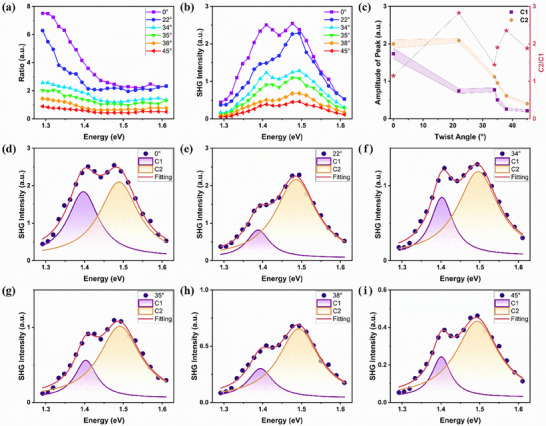
Excitation wavelength‐dependent SHG measurements for TB‐MoS_2_. a) Intensity ratio (η) between bilayer MoS_2_ with different twist angles and monolayer MoS_2_. b) SHG spectra for bilayer MoS_2_ with varying twist angles. c) Amplitude and ratio of the two Lorentzian components. d–i) SHG spectra of TB‐MoS_2_ with twist angles of 0° (d), 22° (e), 34° (f), 35° (g), 38° (h) and 45° (i), respectively. Experimental result expressed as dark blue dots fitted by the Lorentz functions.

Beyond the moiré pattern affecting the PL emission of the as‐grown TB‐MoS_2_, the moiré modulated nonlinear optical properties are extremely sensitive to the SHG spectroscopy.^[^ ^25^, ^49^ ^]^ Here, the wavelength‐dependent SHG measurements were conducted in TB‐MoS_2_ samples using the excitation laser in the energy range of 1.28 to 1.61 eV. The spectra exhibit two resonance peaks, which originate from the contribution of the X^I^.^[^ ^50^ ^]^Previous studies have demonstrated that the SHG intensity is the coherent signal from the two individual MoS_2_ monolayers. The interlayer twist angle introduces a phase difference between two layers, resulting in the SHG intensity described by the equation $I_{t}\left(\theta \right)=I_{1}+I_{2}+2\sqrt{I_{1}I_{2}}\cdot \cos\left(3\theta \right)$, where I_t_, I_1_ and I_2_ represent SHG intensity from the TB system, the top monolayer and the bottom monolayer, respectively, and θ denotes the twist angle.^[^ ^51^ ^]^ Under polarized laser excitation in the absence of a polarizer analyzer before the detector, symmetry arguments dictate that the SHG intensities of the two monolayers satisfy *I*_1_ = *I*_2_  ≡ *I*, This condition reduces the general expression to a simplified form: *I_t_* =  2*I* + 2*I* · cos (3θ). Defining η as the ratio of I_t_ and I (*I_t_*/*I*), the above equation becomes as η  =  2 + 2 · cos(3θ). After considering the interlayer interaction, η is related to the contribution of X^I^, often exhibiting a wavelength‐dependent behavior. To explore this, η as a function of excitation wavelength for bilayers with different twist angles was measured via SHG mapping, using monolayer MoS_2_ as a reference, as shown in **Figure** **4**a, which shows η gradually reduces as the twist angle increases from 0° to 60°. This can be attributed to the decreased SHG interference between the two layers due to the twist angle resulting in the increased phase difference.^[^ ^51^ ^]^ Interestingly, as the excitation energy increases, η gradually drops down, and this trend is similar for all grown TB MoS_2_ at all twist angles. However, this behavior deviates from theoretical expectations based on the simple interference superposition.^[^ ^52^, ^53^ ^]^ as shown in Figure S14 (Supporting Information), suggesting that it is also affected by the resonant coupling of exciton complexes, possibly resulted from the moiré superlattices.^[^ ^52^ ^]^ Similar phenomena have also been found in twisted systems with small twist angles.^[^ ^54^ ^]^ To directly study the X^I^ modulated by twist angle, the relative SHG intensity spectra^[^ ^55^ ^]^ of the as‐grown TB‐MoS_2_ with varying twist angles were determined, as shown in Figure 4b. The spectra clearly reveal two peaks, C_1_ and C_2_, located at ≈1.4 and 1.5 eV, respectively, which reflect the excitons limited by the periodical lattices of the individual layers. The spectra were fitted with Lorentzian functions, as shown in Figure 4d–i. The peak at 1.5 eV (C_2_) is stronger than that at 1.4 eV (C_1_). The intensities of both peaks show a decreasing trend with increasing twist angle, consistent with the overall decreasing trend of SHG intensity observed at larger twist angles. The ratio of C_2_ to C_1_ (C_2_/C_1_) remains largely constant for the most twist angles, as shown in Figure 4c. However, at 22° and 38° twist angles, the C_2_/C_1_ ratio increases sharply, manifesting the enhanced resonant peak at nearly 1.5 eV. This can be ascribed to the single‐photon resonant enhancement of SHG, and the interlayer excitons interacted with the periodical moiré superlattice, which is consistent with the PL results.

## 3 Conclusion

In summary, the high‐quality TB MoS_2_ homostructures with a wide range of twist angles were successfully fabricated via cyclically changing the carrier Ar gas CVD method. For the first time, the growth process of twisted MoS_2_ bilayer guiding by the tilt GBs is clearly unfolded through real‐time monitoring the nucleation and coalesce of the top layer, demonstrating the top layer rotated a twist angle identical to the tilt angle of the GBs. PL spectroscopy investigations on the TB MoS_2_ structures display that the indirect exciton peak shifts in‐red with increasing the twisted angle, which is consistent with the variation of the indirect band gap with the twist angle calculated by PBE theory. In addition, low temperature (10K) PL spectroscopy and SHG results show that interlayer excitons strongly couple with special twist angles in which the intensity of the interlayer exciton can be extremely enhanced in the as‐grown TB‐MoS_2_ at commensurate angles of 22° and 38°. This means that the moiré superlattice provides an effective avenue to tune the interlayer exciton to form a moiré exciton, which may be a universal method to obtain twisted bilayer structures for further tuning the energy band structures and novel spin exciton physics.

## 4 Experimental Section

### CVD Growth of the Top‐Layer Twisted Bilayer MoS_2_ Structures

The top‐layer twisted bilayer MoS_2_ patterns were grown on 300‐nm‐thick SiO_2_/Si substrate by a NaCl‐assisted CVD method, with crystal sizes ranging from 10 to 20 µm. During CVD growth process, MoO_3_ powder (99.9%, 5 mg, Alfa Aesar) and sulfur powder (99.95%, 50 mg, Alfa Aesar) were placed in different temperature zones, meanwhile, the SiO_2_/Si substrate was upside down set away from the MoO_3_ precursor with the distance of 1 cm. Here, the argon gas was used as the carrier gas with the gas flow rate ranging from 100 to 180 sccm for the growth of twisted bilayer MoS_2_ crystals. The furnace temperature was ramped from 30 to 800 °C for 20 min, and the reaction time was kept for 10 min under atmospheric pressure. The temperature of the MoO_3_ and sulfur precursor was set at 800 and 130 °C, respectively. After finishing the MoS_2_ growth, the CVD system was cooled down to room temperature without any assistance.

### Measurement of Raman, and PL Spectroscopy

Low frequency and high frequency Raman spectra were measured at room temperature using a micro‐Raman system (Horiba, LabRAM HR Evolution NANO) equipped with 1800 lines/mm gratings with laser energy of 2.33 eV (532 nm). A ×100 objective lens (numerical aperture = 0.90) was used to focus the laser beam to a spot ≈1 µm in diameter on the sample surface.

PL and LT PL spectra were recorded with a confocal Raman microscope (InVia, Renishaw U.K.) at room temperature with a 532 nm He‐Cd laser as the excitation source. The 100 lines/mm grating was used in the PL measurements.

### Multimodal Second Harmonic Generation Characterization

### Multimodal Second Harmonic Generation Characterization—Polarization‐Dependent SHG Polar Plots

Polar plots of parallel polarized SHG intensity were conducted on a homebuilt microscope system. An 808 nm femtosecond laser (MaiTai HP, Spectra‐Physics; 80 MHz repetition rate) served as the excitation source. The fundamental beam passed through a linear polarizer (GL10, Thorlabs) and a half‐wave plate (WPH10M‐808, Thorlabs) mounted on a rotating stage to control the excitation polarization. The laser was then focused onto the sample using a 40× objective lens (NA 0.6, Olympus), which also collected the backward‐propagating SHG signal from the sample. The SHG signal was reflected by beam‐splitting devices and short‐pass filters (FF01‐451/106 (2) 25, Semrock and FF01‐405/150‐25, Semrock) to suppress residual excitation beam, and analyzed using a rotatable linear polarizer (GL10, Thorlabs) positioned before a photomultiplier tube (PMT, H7422‐40, Hamamatsu). Polarization‐resolved SHG intensity profiles were acquired by synchronously rotating both the excitation half‐wave plate and the detection polarizer.

### Multimodal Second Harmonic Generation Characterization—Laser‐Scanned SHG Imaging

For spatially resolved SHG intensity mapping, a separate laser‐scanning imaging system was employed. The same 808 nm excitation source was used, but the polarization configuration differed: the incident beam was fixed to horizontal linear polarization using a stationary polarizer (GL10, Thorlabs). The laser was raster‐scanned across the sample using a galvanometer scanner (GSV112, Thorlabs) coupled with a scan lens (SL50‐CLS2, Thorlabs) and a tube lens (TL200‐CLS2, Thorlabs). The SHG signal was collected through the same 40× objective, filtered as described above, and detected by the PMT. Two detection modes were utilized: i) polarization‐unresolved imaging without an analyzer, and ii) polarization‐resolved imaging with a linear analyzer (GL10, Thorlabs) to assess orientation‐dependent signal contributions.

### Multimodal Second Harmonic Generation Characterization—Wavelength‐Dependent SHG Spectroscopy

Spectral characterization of the SHG signal was conducted using the laser‐scanning imaging system described in Section 2. The excitation wavelength was tuned across 770–970 nm while maintaining horizontal linear polarization. The SHG signal was collected without an analyzer to maximize intensity, spectrally isolated using the same short‐pass filters, and recorded by the PMT to generate wavelength‐dependent intensity profiles.

## Conflict of Interest

The authors declare no conflict of interest.

## Author Contributions

X. T. L. and X. Z. have equal contributions to this work. R.F.D. and X.T.L. conceived the present experiments. X.T.L. prepared the samples of MoS_2_ bilayer on SiO_2_/Si substrates. X. Z. and W.K.Z performed the SHG experiments. X.T.L, Z.H.Z., and N. L. carried out the Raman and PL spectroscopy measurements. R.F.D., X.T.L., T.T.Z, and J. Y. analyzed the experimental data and calculated data. L.N.Z. and J. F. G carried out the DFT calculation of the energy band structures of TB MoS_2_ homostructures. R.F.D. completed writing the text. All authors discussed the results and commented on the manuscript.

## Supporting information



Supporting Information

Supplemental Movie 1

Supplemental Movie 2

Supplemental Movie 3

## Data Availability

The data that support the findings of this study are openly available from the corresponding author (Dr. Ruifen Dou) upon reasonable request.
