# Supplementary material for: Controlling Twisted Angles in Directly Grown MoS2 Bilayers via Tilt Grain Boundary Engineering
Source: Adv Sci (Weinh). 2025 Aug 22;12(42):e09280. doi: 10.1002/advs.202509280 (PMC12622490; doi:10.1002/advs.202509280)
Supplement: Supplementary file 1 — Supporting Information [file ADVS-12-e09280-s004.docx]

**Supporting Materials**

**Controlling Twisted Angles in Directly Grown MoS₂ Bilayers via Tilt Grain Boundary Engineering**

Xiaotian Li,^1,#^ Xuan Zhao,^1,4,#^ Luneng Zhao,^2^ Junfeng Gao,^2^ Zihan Zhao,^3^ Jian Yang,^1^ Tiantian Zhang,^1^ Yibiao Feng,^1^ Zejun Luo,^1^ Nan Liu,^3^ Jiacai Nie^1^, Wenkai Zhang,^1,4,✉^ Ruifen Dou^1,✉^

^1^ Key Laboratory of Multiscale Spin Physics, Ministry of Education, School of Physics and Astronomy, Beijing Normal University, Beijing, 100875, P. R. China

^2^ Lab Material Modification Laser Ion & Electron Beams, Dalian University of Technology, Dalian 116024, P. R. China

^3^ Beijing Key Laboratory of Energy Conversion and Storage Materials, College of Chemistry, Beijing Normal University, Beijing 100875, P. R. China

^4^ Applied Optics Beijing Area Major Laboratory, Center for Advanced Quantum Studies, School of Physics and Astronomy, Beijing Normal University, Beijing 100875, P.R. China

Corresponding Authors: [rfdou@bnu.edu.cn](mailto:rfdou@bnu.edu.cn); [wkzhang@bnu.edu.cn](mailto:wkzhang@bnu.edu.cn).

**Table of Contents:**

[**Figure S1. (a-s) Optical microscope images of TB MoS_2_.** 3](#_Toc206020773)

[**Figure S2. Scanning electron microscopy (SEM) images of TB MoS_2_ samples.** 4](#_Toc206020774)

[**Figure S3. The verification of GBs location and growth mechanism in TB MoS_2_.** 5](#_Toc206020775)

[**Figure S4. The fundamental characterizations of SHG signal including wavelength verification and power dependence analysis.** 6](#_Toc206020776)

[**Figure S5. Polarization-resolved SHG to calibrate the twist angle of TB MoS_2_.** 7](#_Toc206020777)

[**Table S1. The relationship of the twist angle of the two layers and the tilt angle of the two twin grains.** 8](#_Toc206020852)

[**Figure S6. Polarization-resolved SHG intensity mapping under excitation at λ_ex_ = 808 nm for 22° (a), 35° (b), 38° (c) and 45° (d) TB MoS_2_.** 9](#_Toc206020778)

[**Figure S7. Polarization-resolved SHG analysis of strain at grain boundaries.** 10](#_Toc206020779)

[**Table S2. The compressive deformation in the six grain boundary regions.** 10](#_Toc206020853)

[**Note 1: strain field model based on photoelastic tensor theory** 11](#_Toc206020733)

[**Figure S8. Grain boundary characterization in a star-shaped MoS_2_ flake.** 12](#_Toc206020780)

[**Figure S9. The schematic model and in-time visualization of the growth process for the upper layer on the multi-twin grain composed bottom layer.** 12](#_Toc206020781)

[**Note 2: Twist-Angle-Dependent Phonon Properties in TB MoS_2_ Probed by Raman Spectroscopy** 13](#_Toc206020734)

[**Figure S10. Determination of the twist angles and the interlayer interaction of twisted MoS_2_ bilayer at a wide range of twist angles.** 14](#_Toc206020782)

[**Figure S11. Atomic and PBE band structures of twisted bilayer MoS_2_.** 15](#_Toc206020783)

[**Figure S12. (a) Schematic illustration of the moiré superlattice formed in a TB MoS_2_ with 21.8° in real space, and the moiré potential period labeled as a_M_.** 16](#_Toc206020784)

[**Figure S13. Low-temperature PL spectra of the Gaussian fitted bilayer MoS_2_ with twist angles of (a) 25°, (b) 33°, (c) 34°, (d) 41°, (e) 42° and (f) 45°.** 17](#_Toc206020785)

[**Table S3. The intensity of X^A-^, X^IE^, X^A0^ in low-temperature (10K) PL spectra for 22°, 38°, 47° and 60° TB-MoS_2_ and the ratio between them.** 18](#_Toc206020854)

[**Figure S 14. The ratio of SHG intensity between bilayer and monolayer TB-MoS_2_ with different twist angles.** 19](#_Toc206020786)

**
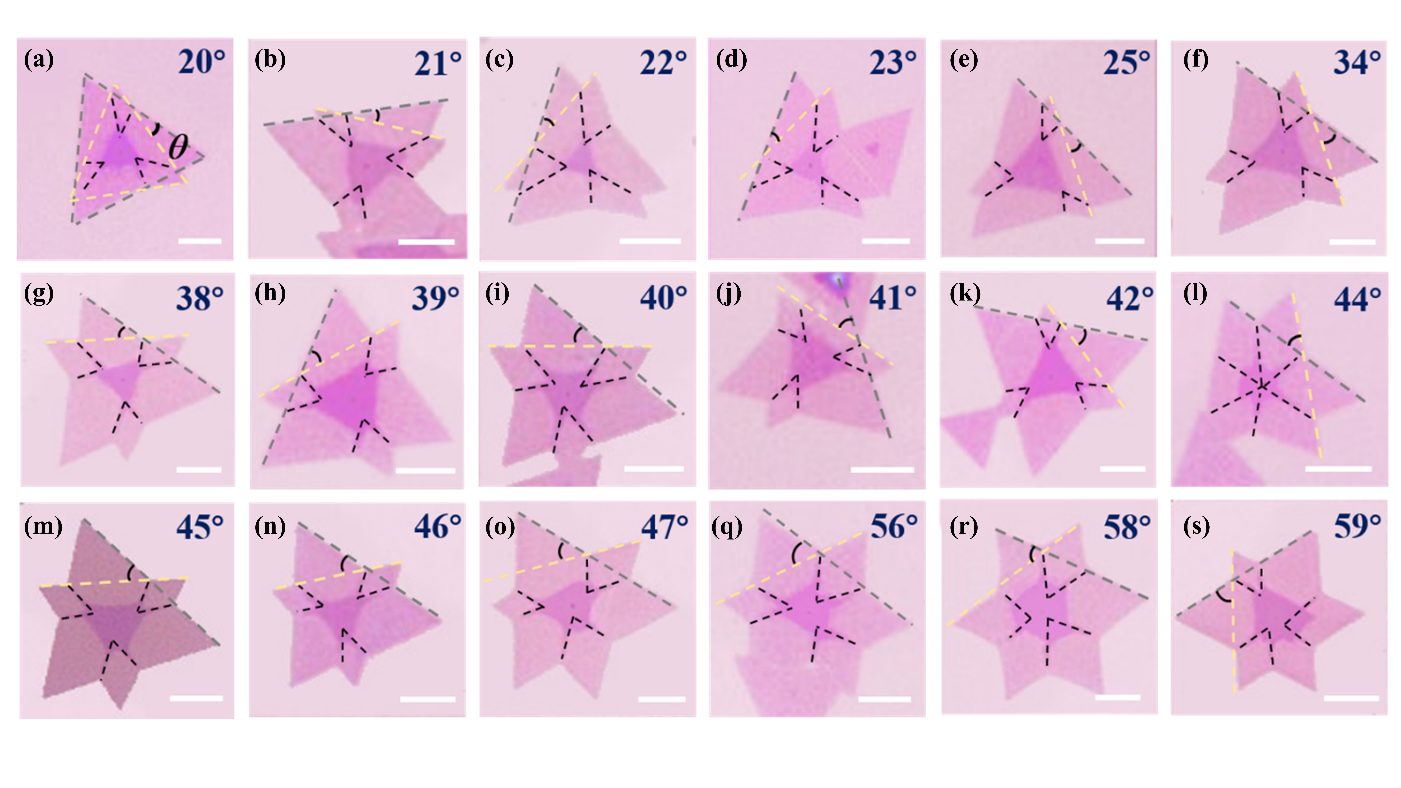
**

**Figure S1. (a-s) Optical microscope images of TB MoS_2_.**

(The edge of the bottom layer is traced by grey dashed line, and the edge of the top layer is traced by yellow dashed line). The defined twisted angle between the top and the bottom layer as θ is shown in optical image of **a**. The scare bars in OM images are all 2 μm.


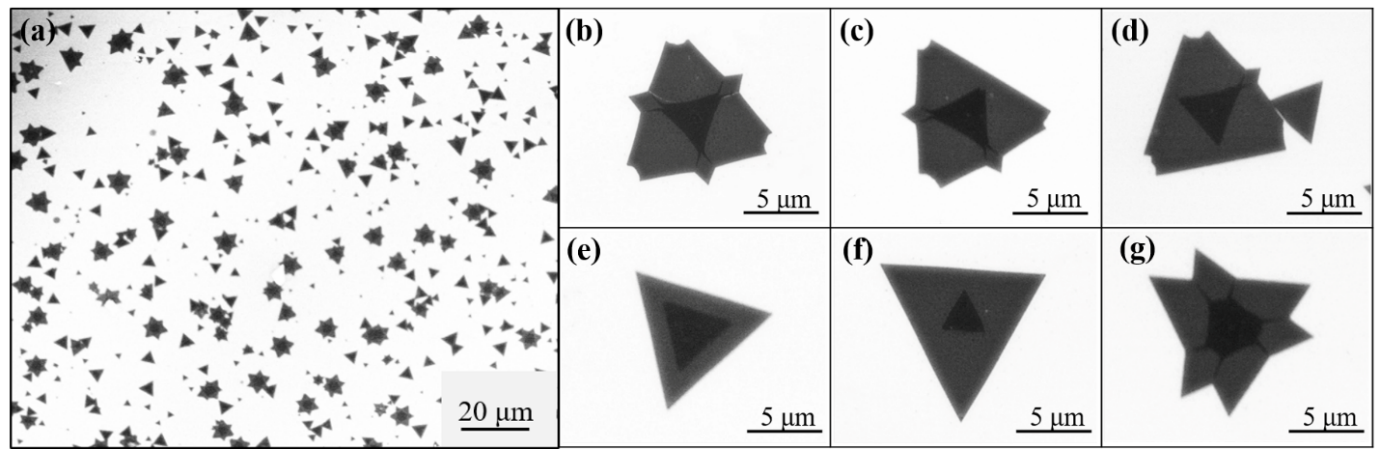


**Figure S2. Scanning electron microscopy (SEM) images of TB MoS_2_ samples.**

(a) Large-scale view of directly synthesized TB MoS_2_ crystals on a SiO_2_/Si substrate. (b-d) Representative 60°-TB MoS_2_ structures formed by the top triangle layer and bottom layer with (b) hexagonal, (c) pentagonal and (d) tetragonal polycrystalline morphologies. (e-g) Bilayer MoS_2_ crystals exhibiting distinct stacking geometries, (e) AA-stacked triangle bilayer (0° twist), (f) AB-stacked triangular bilayer (60° twist), and (g) 33°-TB MoS_2_, where the twist angle is equal to the tilt angle of the bottom coalesced crystals.

**
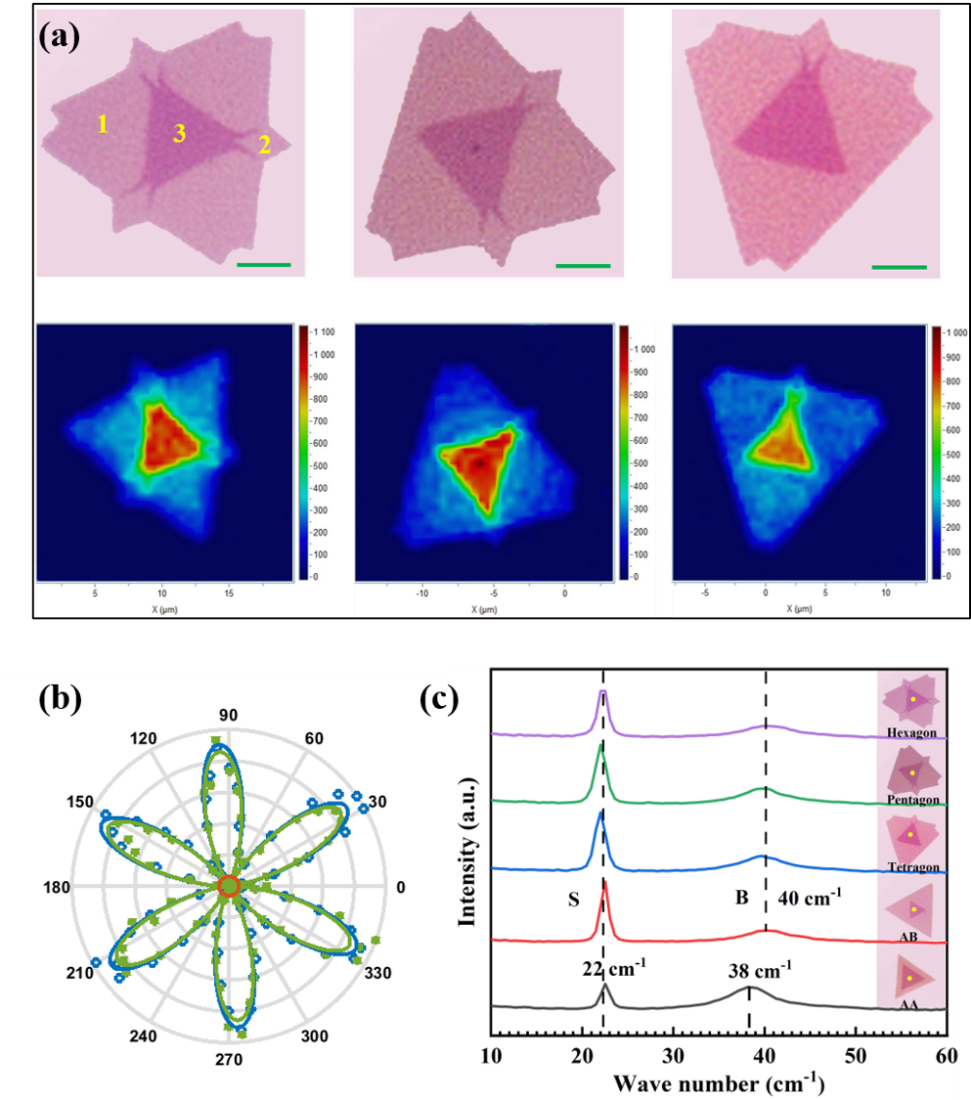
**

**Figure S3. The verification of GBs location and growth mechanism in TB MoS_2_.**

(a) Optical microscope images and corresponding Raman E^1^_2g_ intensity mapping of representative TB MoS_2_. The scale bar is 3um. (b) The polarization-dependent SHG response of the hexagonal MoS_2_ bilayer. Data from distinct regions (labeled 1, 2, and 3 in the inset optical image) are plotted in blue, green, and orange, respectively (c) The low-frequency (LF) Raman spectra of the hexagonal, pentagonal, and tetragonal stacks with a bottom tilt angle of 60°, triangular AA and AB stackings. Corresponding optical microscopy images are shown to the right, with yellow areas indicating the measurement locations.


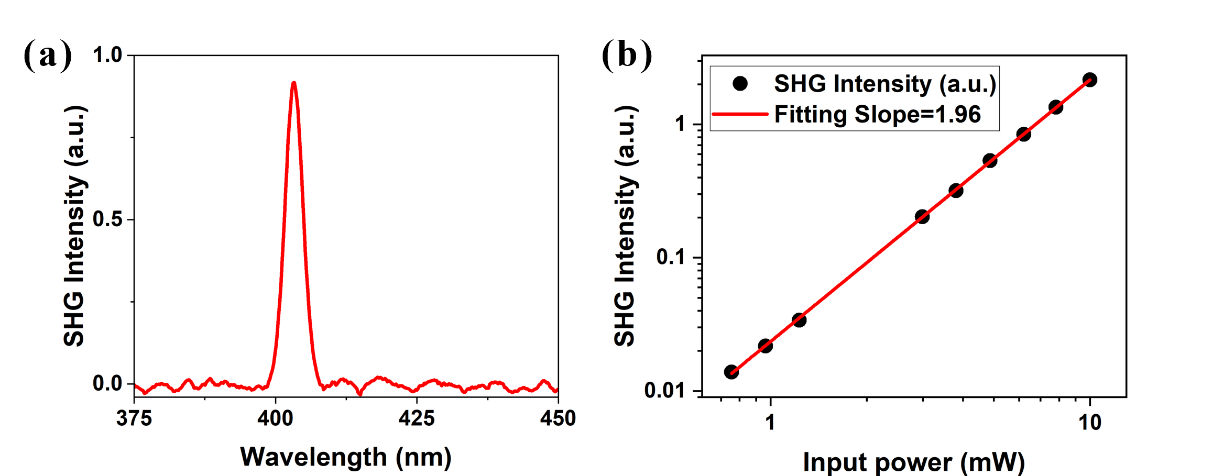


**Figure S4. The fundamental characterizations of SHG signal including wavelength verification and power dependence analysis.**

(a) The SHG spectrum of TB MoS_2_ with a prominent peak at 404 nm, excited at λ_ex_=808 nm. (b) The double-logarithmic plot of power-dependent SHG intensity for TB MoS_2_. The experimental data is well-fitted by the equation $y=aX^{b}$, with the fitted 𝑏 value of 1.96, consistent with a second-order nonlinear optical process.


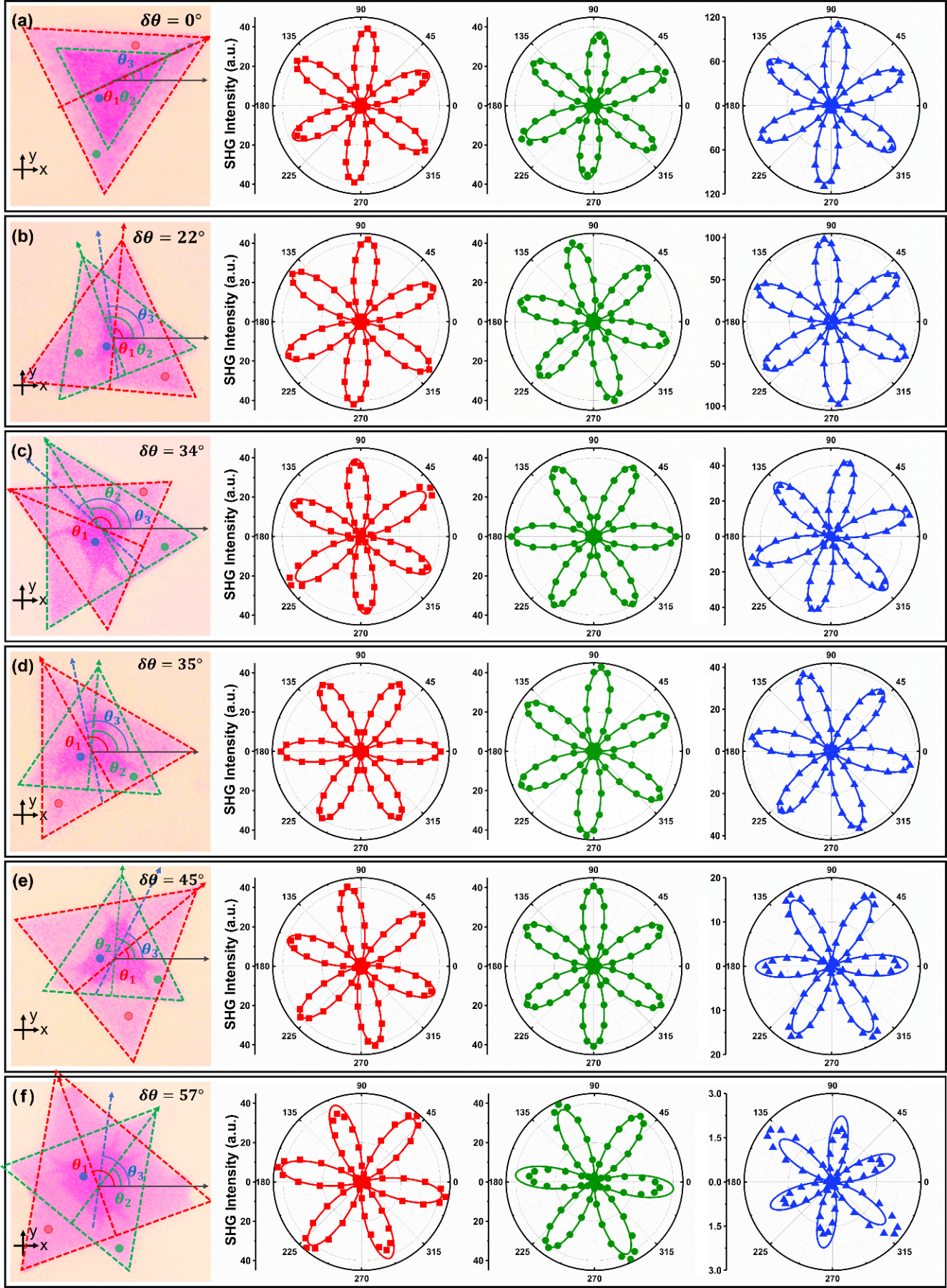


**Figure S5. Polarization-resolved SHG to calibrate the twist angle of TB MoS_2_.**

Polar plots of the parallel components of SHG intensity for TB MoS₂ with twist angles of 0° (a), 22° (b), 34° (c), 35° (d), 45° (e), and 57° (f). Raw data and fitted curves for monolayer 1 (red), monolayer 2 (green), and TB MoS_2_ (blue) are shown, respectively.

**Table S1. The relationship of the twist angle of the two layers and the tilt angle of the two twin grains.**


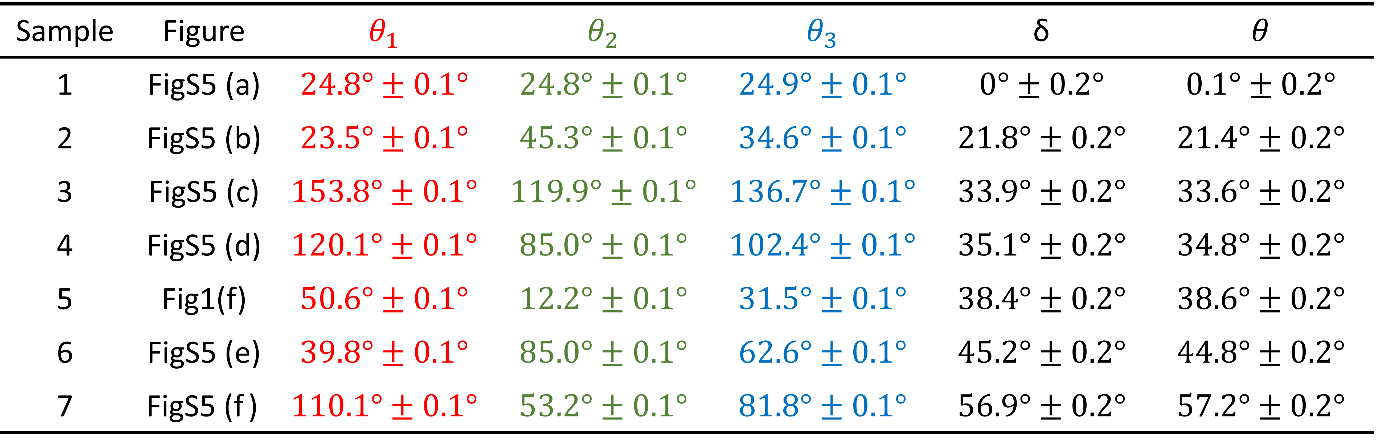


The orientation and twist angle of the samples in FigS5 and Fig1 were calibrated by polarization-resolved SHG. $\boldsymbol{\theta}_{\boldsymbol{1}}$ is the armchair orientation of monolayer1, $\boldsymbol{\theta}_{\boldsymbol{2}}$ is the armchair orientation of monolayer2, $\boldsymbol{\theta}_{\boldsymbol{3}}$ is the orientation of the bilayer's two-fold symmetry axis. $\boldsymbol{\theta}$ is the twist angle between the two layers and $\boldsymbol{\delta}$ is the tilt angle between the two twin grains. The above angle theory has the following relationship:

$$\boldsymbol{\theta=2\times}\left| \boldsymbol{\theta}_{\boldsymbol{1}}\boldsymbol{-}\boldsymbol{\theta}_{\boldsymbol{3}} \right|\boldsymbol{=2\times}\left| \boldsymbol{\theta}_{\boldsymbol{2}}\boldsymbol{-}\boldsymbol{\theta}_{\boldsymbol{3}} \right|\boldsymbol{, \delta=}\left| \boldsymbol{\theta}_{\boldsymbol{1}}\boldsymbol{-}\boldsymbol{\theta}_{\boldsymbol{2}} \right|$$

The above SHG results further provide direct evidence of the interlayer twist angle (θ) is coincident with the tilt angle (δ) between two twin grains in the bottom layer.


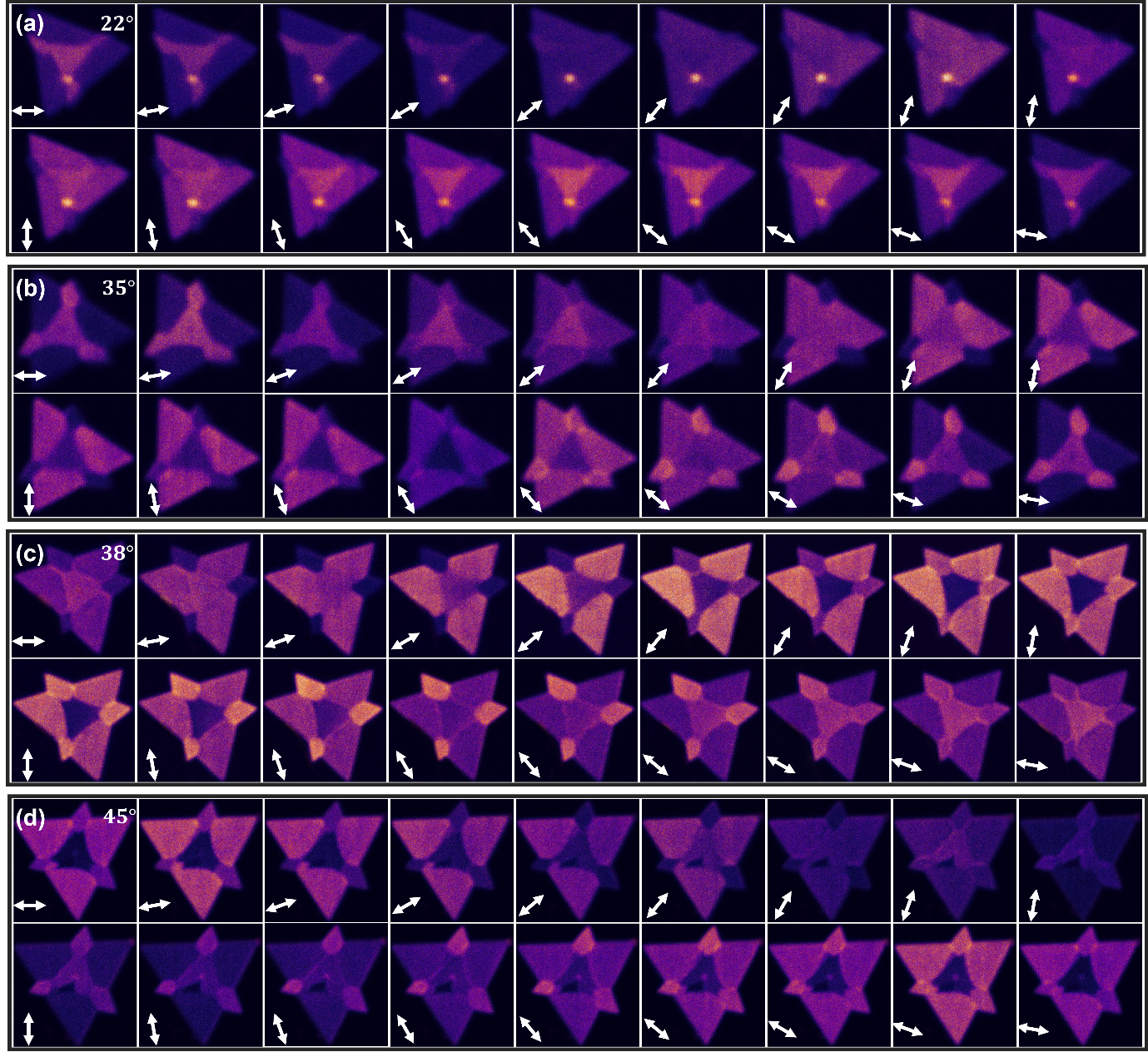


**Figure S6. Polarization-resolved SHG intensity mapping under excitation at λ_ex_ = 808 nm for 22° (a), 35° (b), 38° (c) and 45° (d) TB MoS_2_.**

Arrows indicate the analyzer orientation. Rotating the analyzer (ϕ∈[0°–170°], in 10° steps) reveals the SHG signal switching on and off in different regions, depending on their relative armchair orientation.

**Dynamic videos showcasing the polarization-resolved SHG intensity mapping of the TB-MoS_2_ crystal are available in supplementary material.**


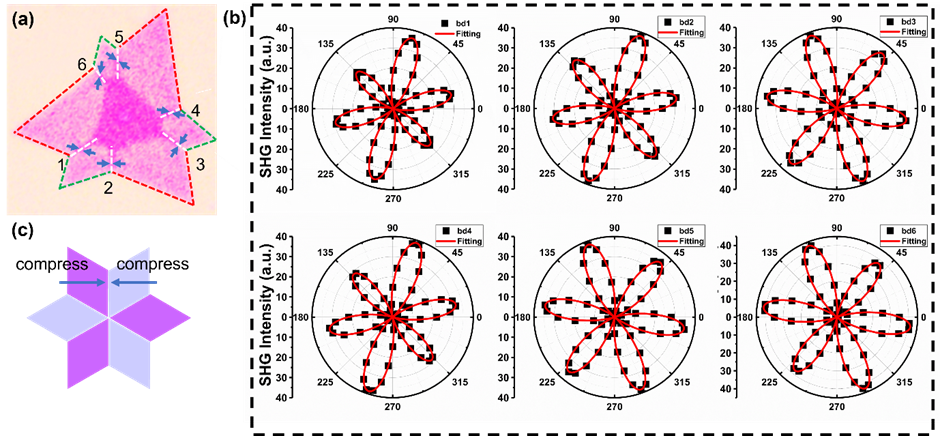


**Figure S7. Polarization-resolved SHG analysis of strain at grain boundaries.**

(a) OM image of TB-MoS₂. White dashed lines indicate grain boundary locations, with blue arrows showing the directions of compressive deformation. Numbers 1–6 label the grain boundaries. (b) Polar plot of the parallel components of SHG intensity for six grain boundaries. Black squares represent raw data, and red curves show the fitting results based on the strain field model analyzed using photoelastic tensor theory. (c) Schematic diagram of compressive deformation within the grain boundary region.

**Table S2. The compressive deformation in the six grain boundary regions.**


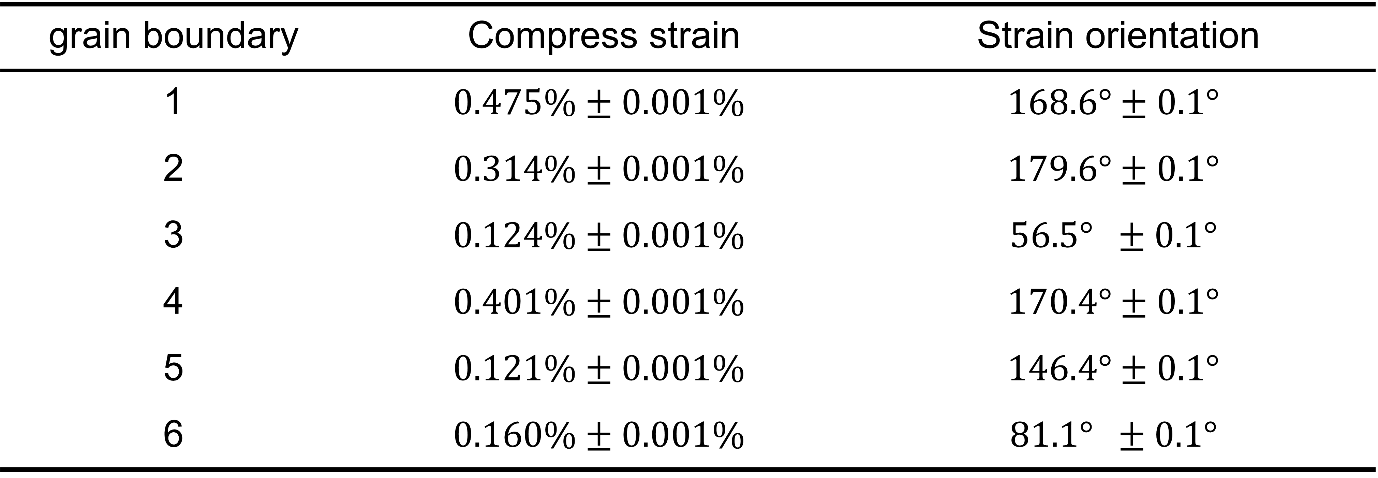


**Note 1: strain field model based on photoelastic tensor theory**

The Polar plot of the parallel components of SHG intensity is given by:

$$I_{para}^{2}\left( 2\omega\right)\propto\frac{1}{4}\left( Acos\left( 3\phi-3\phi_{0} \right)+B\left( 2\theta+\phi-\phi_{0} \right) \right)^{2}$$

Where:

$A=\left( 1-\nu\right)\left( p_{1}+p_{2} \right)\cdot\varepsilon_{ua}+2\chi_{0}$

$B=\left( 1+\nu\right)\left( p_{1}-p_{2} \right)\cdot\varepsilon_{ua}$

Definitions:

$p_{1}$ and $p_{2}$ : Photoelastic parameters

$\varepsilon_{ua}$: Uniaxial strain

$\theta$: Principal strain orientation

$\phi$ and $\phi_{0}$ : Polarization angle and the armchair orientation of the sample

$\chi_{0}$: Nonlinear susceptibility parameter of the unstrained crystal lattice

Based on previous studies^1^, the parameters are as follows:

$\nu=0.29$

$\chi_{0}=4.5nm/V$

$p_{1}=-0.68nm/V/\%$

$p_{2}=-2.35nm/V/\%$.

For compressive strain, $\varepsilon_{ua}<0$.


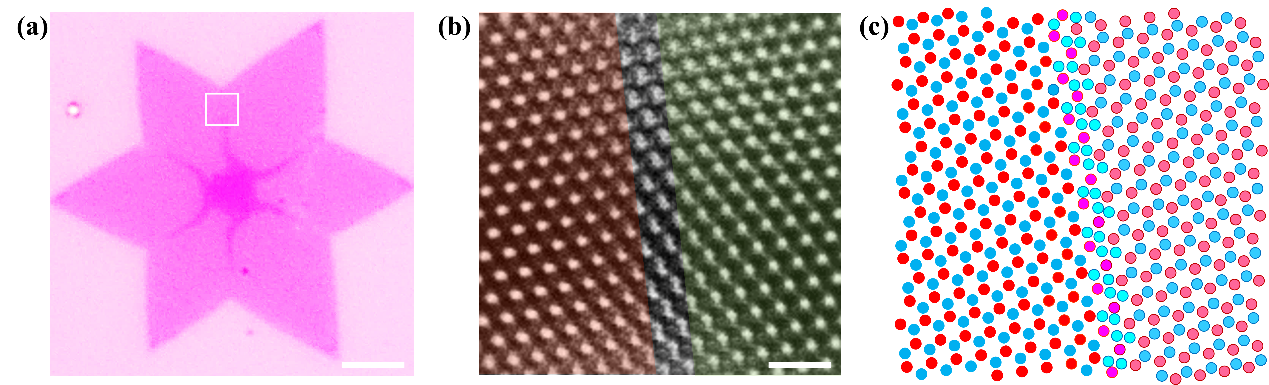


**Figure S8. Grain boundary characterization in a star-shaped MoS_2_ flake.**

(a) OM image of a representative star-shaped MoS_2_ crystal. The location of the analyzed grain boundary is indicated (e.g., white box). The scale bar is 5μm (b) High-resolution HAADF-STEM image acquired at the grain boundary region identified in (a), resolving the atomic arrangement at the interface. Distinct domains are colored (red and green). The scale bar is 1nm. (c) Schematic representation of the atomic structure of the grain boundary observed in the HAADF-STEM image (b).


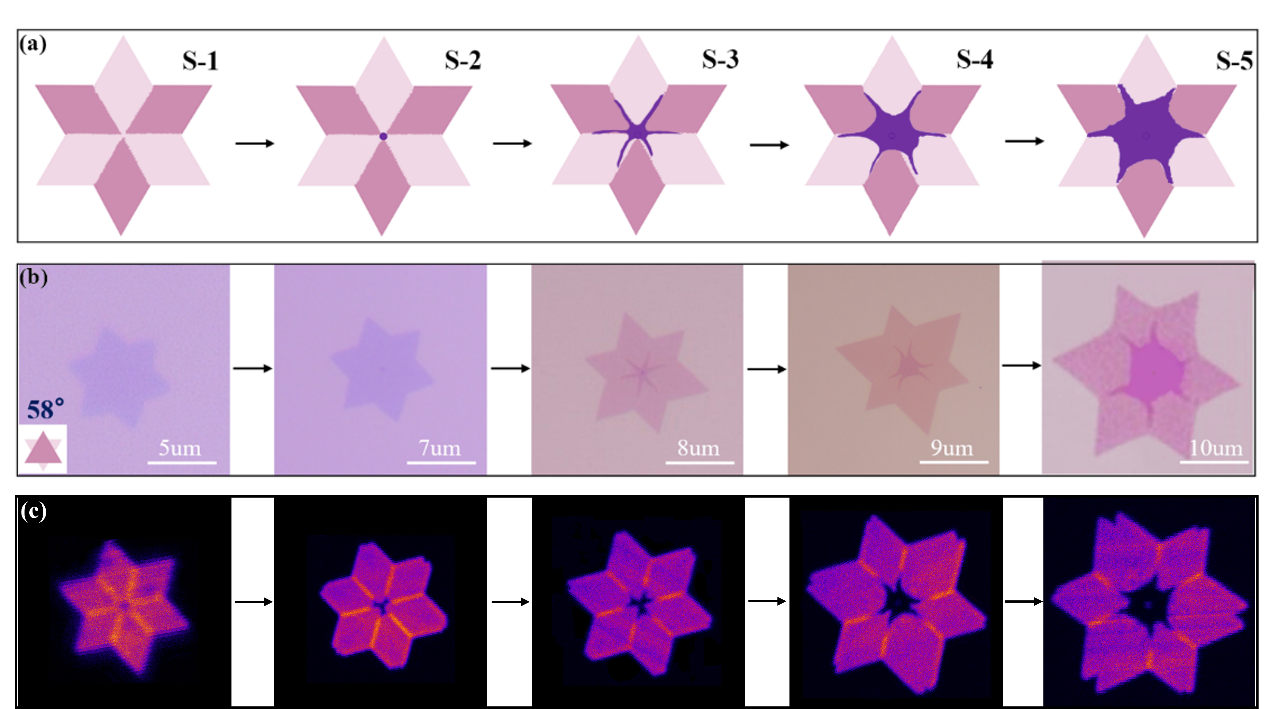


**Figure S9. The schematic model and in-time visualization of the growth process for the upper layer on the multi-twin grain composed bottom layer.**

**(a)** A serial of schematic modes showing the step-by-step growth process for the nucleation and growth of the second layer guided by the tilt boundary in the first layer. **(b-c)** Real time monitoring the growth process of the second layer in the top of the first layer by OM images and corresponding SHG intensity mapping images.

**Note 2: Twist-Angle-Dependent Phonon Properties in TB MoS_2_ Probed by Raman Spectroscopy**

To verify the crystal quality of the as-grown TB MoS_2_, Raman spectroscopy is further performed for all as-grown MoS_2_ with a wide range of angles. The low-frequency (LF) and high-frequency (HF) Raman spectra are shown in Figure 3a and b, respectively, proving that Raman vibration modes are strongly dependent on twist angles. Firstly, the LF Raman spectra are sensitive to the layer numbers and interlayer coupling in TMD materials, allowing for effective reflection of changes in interfacial coupling of van der Waals structures. The LF Raman spectrum of 60°-MoS_2_ bilayer showing a shear (S) mode at 22.3 cm^-1^ and the breath (B) mode at 40.2 cm^-1^. Compared to the LF spectrum of 60°-MoS_2_ bilayer, the energy positions of the S mode and the B mode almost have no obvious change for 56°-TB MoS_2_ structure, except for the intensity of the S mode degrading. However, the LF Raman spectra from the TB MoS_2_ with the twist angle larger than 20° but exception of 56° show that the S mode disappears and the B mode shifts to the low energy of about 35.5 cm^-1^. This phenomenon suggests the interlayer coupling can be tuned with the introduction of a twist angle, which is in agreement with the previous findings on twisted bilayer structures.^2-4^ HF Raman spectra of TB MoS2 at different twist angles in Figure 3b shows two prominent peaks located at approximate 384 cm^-1^ and 404 cm^-1^, which can be respectively ascribed to the out of $E_{2g}^{1}$ mode and the in-plane $A_{1g}$mode of the MoS_2_ crystal. Compared to 0° (60°)-MoS_2_ bilayer, the $A_{1g}$mode shifts a bit to the low frequency, while the $E_{2g}^{1}$ mode remains stable with the interlayer twists increasing from 0° to 30°, and from 30° to 60°. The $A_{1g}$ mode mainly involves interlayer vibrations of S atoms, and thus the slight red shift in the $A_{1g}$ mode can be attributed to the weakened long-range Coulombic interlayer interaction induced by the large interlayer twists. This result is in compatible with the LF Raman results. It is worth noting that a peak at around 410 cm^-1^ is identified in HF Raman curves for the TB MoS_2_ with twist angles ranging from 20° to 47°, which can be ascribed to the folded phonon vibration modes (${FA}_{1g}$).^1,4^ Meanwhile, Figure 3c further show that ${FA}_{1g}$peaks exhibit a sine-like behavior related to interlayer twist angles, in agreement with the previous studies on moiré phonon modes.^2^ Besides, the normalized ${FA}_{1g}$ peak in Figure 3d shows a significant enhancement in the intensity of ${FA}_{1g}$for 22°- and 38°-TB MoS_2_ homostructures, which is probably due to these two twist angles almost near to the commensurate angles of 21.8° and 38.2° and thus resulting in the periodical moiré superlattices. The moiré phonons in moiré superlattices with the commensurate angles of 22° and 38° stem from diverse wave vectors of phonon dispersion, further generating the phonon-assisted electron excited states in K space. Therefore, moiré superlattice not only provides an effective manner to manipulate the phonon dispersion, but also tune the energy band structures and the resulting optical properties.


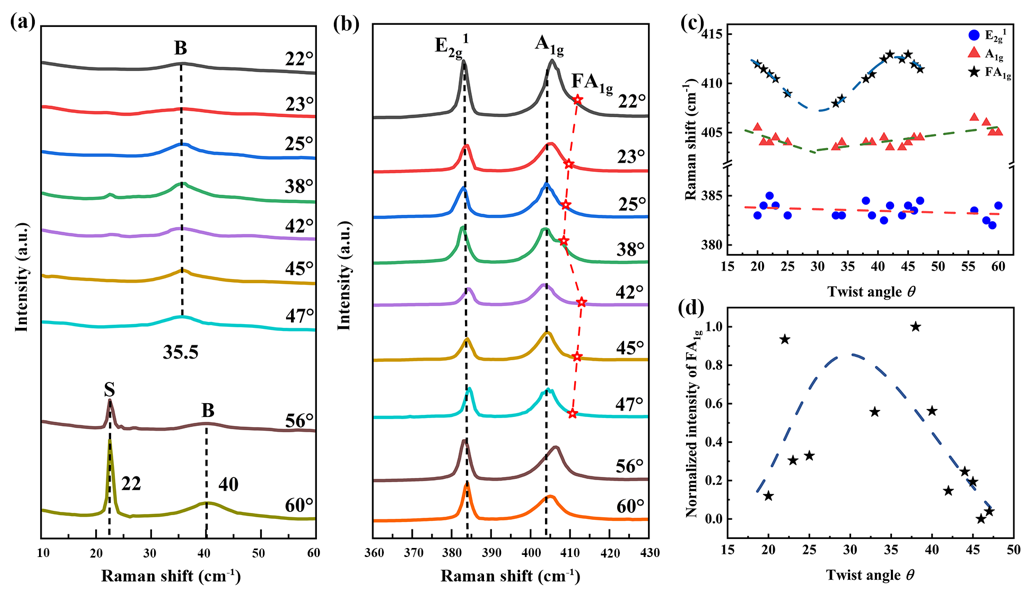


**Figure S10. Determination of the twist angles and the interlayer interaction of twisted MoS_2_ bilayer at a wide range of twist angles.**

**(a)** LF Raman spectra of bilayer MoS_2_ with a wide range of twist angles. S and B represent the shear mode and the breathing mode, respectively. **(b)** HF Raman spectra of twisted bilayer corresponding to (a). The vertical dashed lines are the guide lines for the peaks of $E_{2g}^{1}$ and $A_{1g}$, and the red pentagram represents the folded moiré phonon (${FA}_{1g}$) peak. **(c)** The position of $E_{2g}^{1}$, $A_{1g}$, and ${FA}_{1g}$ Raman peaks as a function of twist angle. **(d)** Normalized intensity of ${FA}_{1g}$peak of twisted bilayer MoS_2_.


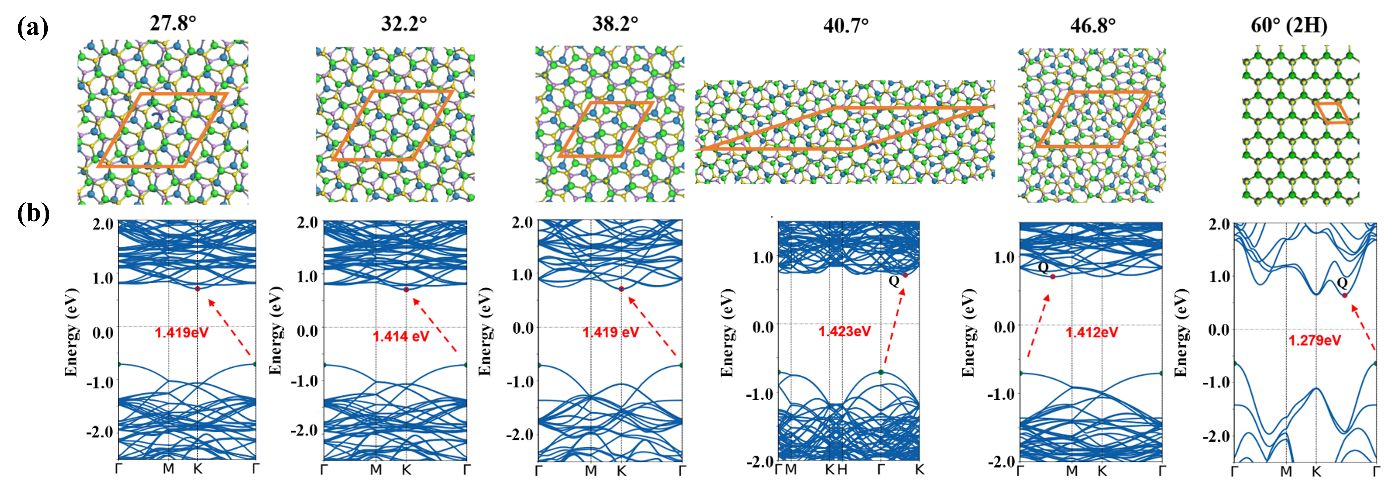


**Figure S11. Atomic and PBE band structures of twisted bilayer MoS_2_.**

(a) The geometrical structure of twisted MoS_2_ bilayers with angles of 27.8°, 32.2°, 38.2°, 40.7°, 46.8° and 60°, respectively. The blue and purple balls in the geometrical structures represent the Mo atom and S atom for the underlying MoS_2_ monolayer and the green and yellow balls represent the Mo atom and S atom for the upper MoS_2_ monolayer. The orange regions represent the moiré supercells in twisted MoS_2_ bilayers. (b) Electronic band structure of twisted MoS_2_ bilayers with angles of 27.8°, 32.2°, 38.2°, 40.7°, 46.8° and 60°, respectively calculated with the PBE.

**
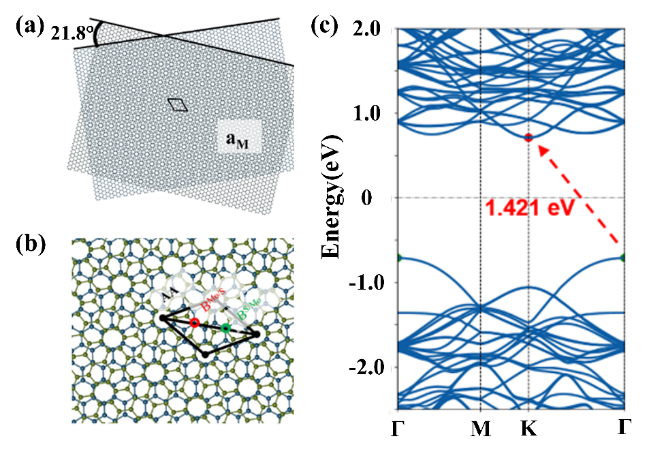
**

**Figure S12. (a) Schematic illustration of the moiré superlattice formed in a TB MoS_2_ with 21.8° in real space, and the moiré potential period labeled as a_M_.**

**(b)** The moiré superlattice in **(a)** leads to periodic modulation of the electrostatic potential of the twisted homobilayer, showing three highlighted regions with the three-fold rotational symmetry (AA, B^Mo/S^, B^S/Mo^), which may retain excitons and complexes of exciton. **(c)** Electronic band structure of twisted MoS_2_ bilayers with angles of 21.8° calculated with the PBE.

**
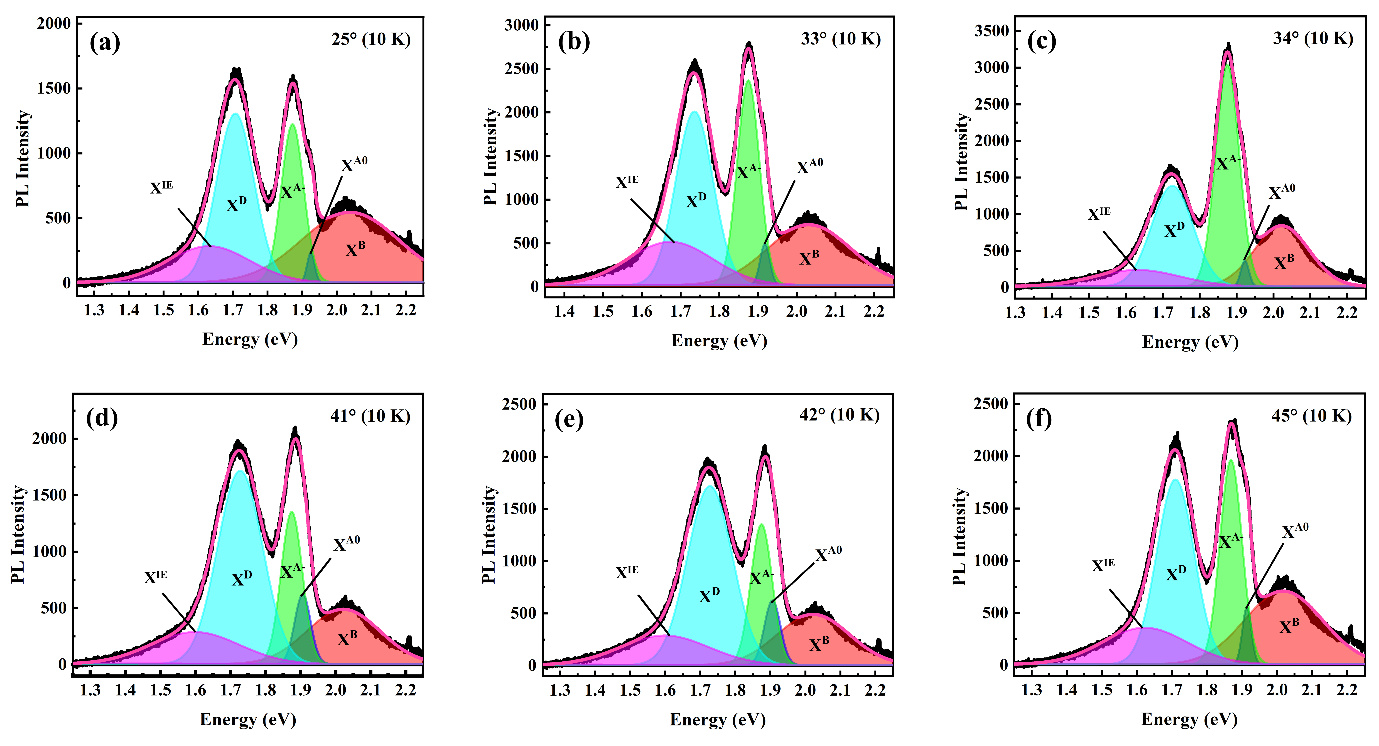
**

**Figure S13. Low-temperature PL spectra of the Gaussian fitted bilayer MoS_2_ with twist angles of (a) 25°, (b) 33°, (c) 34°, (d) 41°, (e) 42° and (f) 45°.**

**Table S3. The intensity of X^A-^, X^IE^, X^A0^ in low-temperature (10K) PL spectra for 22°, 38°, 47° and 60° TB-MoS_2_ and the ratio between them.**

**
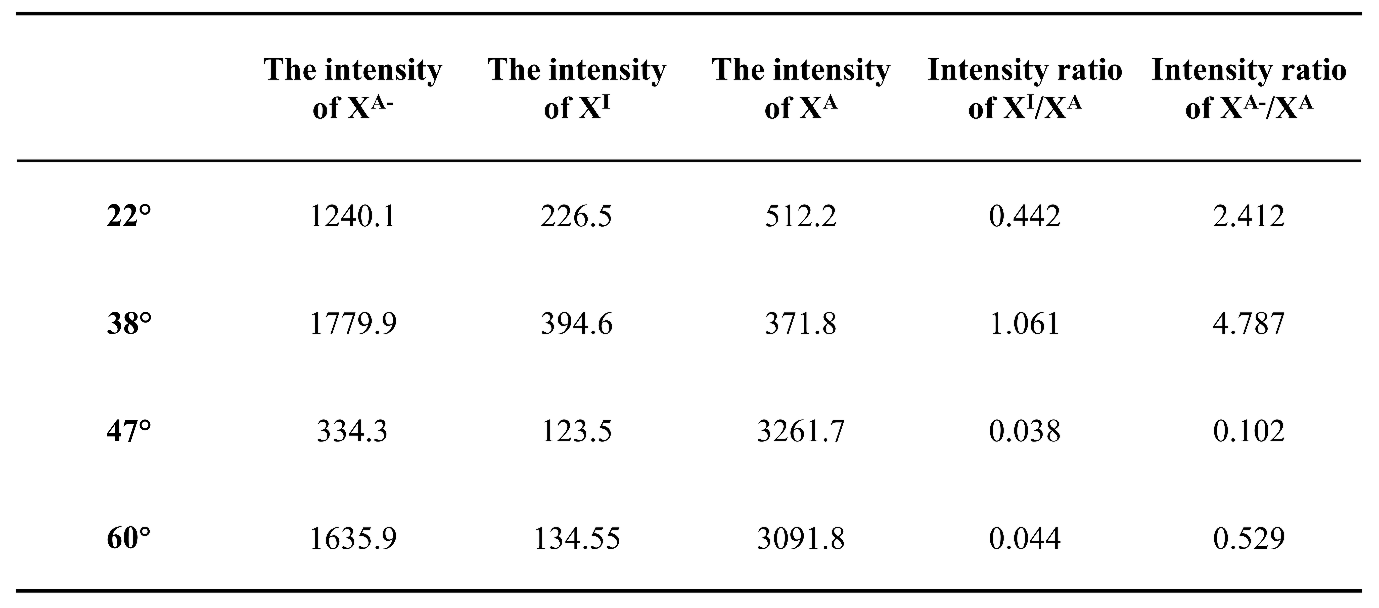
**


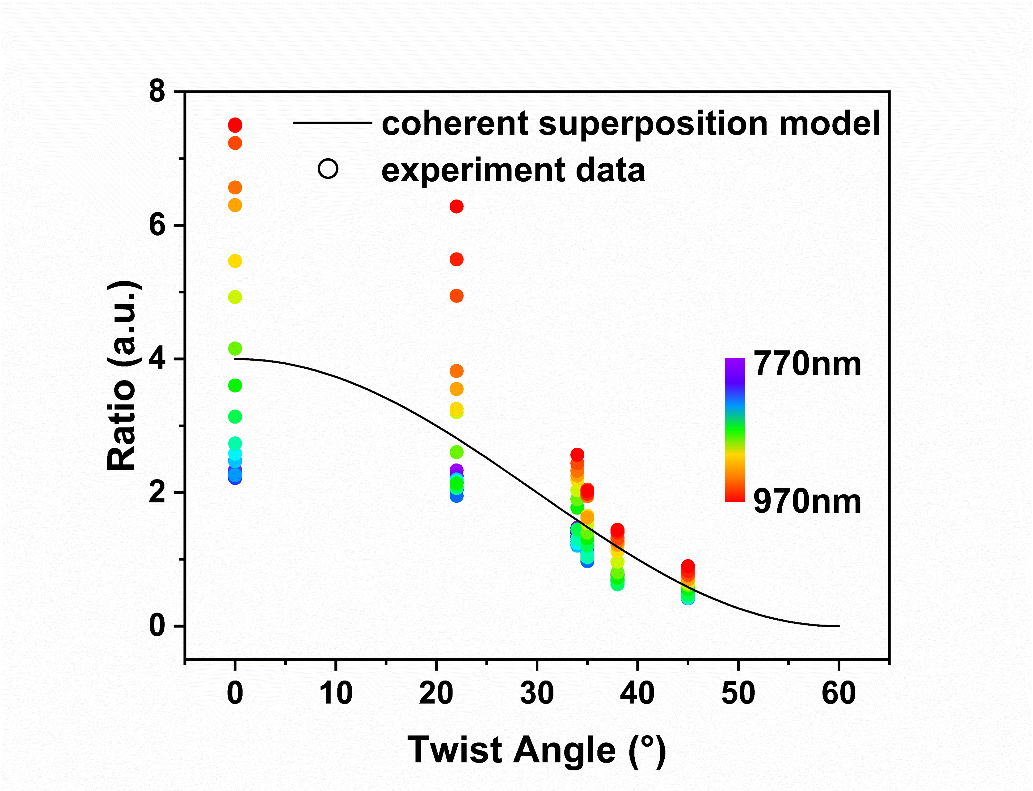


**Figure S 14. The ratio of SHG intensity between bilayer and monolayer TB-MoS_2_ with different twist angles.**

**References**

1. L. Mennel, M. M. Furchi, S. Wachter, M. Paur, D. K. Polyushkin, T. Mueller, Optical imaging of strain in two-dimensional crystals. *Nature Communications*, **9**, 516 (2018).
2. X. Zhang, W. P. Han, J. B. Wu, S. Milana, Y. Lu, Q. Q. Li, A. C. Ferrari, P. H. Tan, Raman spectroscopy of shear and layer breathing modes in multilayer MoS_2_. *Phys. Rev. B.* **87**, 115413 (2013).
3. L. Liang, J. Zhang, B. G. Sumpter, Q.-H. Tan, P.-H. Tan, V. Meunier, Low-Frequency Shear and Layer-Breathing Modes in Raman Scattering of Two-Dimensional Materials. *ACS Nano* **11**, 11777-11802 (2017).
4. M. L. Lin, Q. H. Tan, J. B. Wu, X. S. Chen, J. H. Wang, Y. H. Pan, X. Zhang, X. Cong, J. Zhang, W. Ji, P. A. Hu, K. H. Liu, P. H. Tan, Moiré Phonons in Twisted Bilayer MoS_2_. *ACS Nano* **12**, 8770-8780 (2018).
5. B. Wang, M. Huang, N. Y. Kim, B. V. Cunning, Y. Huang, D. S. Qu, X. J. Chen, S. Jin, M. Biswal, X. Zhang, S. H. Lee, H. Lim, W. J. Yoo, Z. Lee, R. S. Ruoff, Controlled Folding of Single Crystal Graphene. *Nano Lett.* **17**, 1467-1473 (2017).
